# Microbiome diversity protects against pathogens by nutrient blocking

**DOI:** 10.1126/science.adj3502

**Published:** 2023-12-15

**Authors:** Frances Spragge, Erik Bakkeren, Martin T. Jahn, Elizete B. N. Araujo, Claire F. Pearson, Xuedan Wang, Louise Pankhurst, Olivier Cunrath, Kevin R. Foster

**Affiliations:** 1 Department of Biology, University of Oxford, Oxford, UK; 2 Department of Biochemistry, University of Oxford, UK; 3 Kennedy Institute of Rheumatology, University of Oxford, UK; 4 CNRS, UMR7242, Biotechnology and cell signaling, University of Strasbourg, Illkirch, France

## Abstract

The human gut microbiome plays an important role in resisting colonization of the host by pathogens, but we lack the ability to predict which communities will be protective. We studied how human gut bacteria influence colonization of two major bacterial pathogens, both *in vitro* and in gnotobiotic mice. While single species alone had negligible effects, colonization resistance greatly increased with community diversity. Moreover, this community-level resistance rested critically upon certain species being present. We explain these ecological patterns via the collective ability of resistant communities to consume nutrients that overlap with those used by the pathogen. Further, we apply our findings to successfully predict communities that resist a novel target strain. Our work provides a reason why microbiome diversity is beneficial and suggests a route for the rational design of pathogen-resistant communities.

## Introduction

The human gut is home to diverse bacterial species collectively known as the gut microbiota. A major health benefit provided by the gut microbiota is protection against pathogen colonization and subsequent infection; a phenomenon known as colonization resistance (1). The ability of the microbiota to protect against numerous enteric pathogens is well-documented, with evidence that particular species within the microbiota play a more important role than others (2–9). The ways that colonization resistance can arise include competition for nutrients and space, direct antagonism by toxins and other harmful compounds, and promoting host immunity against pathogens (1, 10, 11).

While the importance of the microbiota for colonization resistance is clear, however, we currently lack the principles needed to predict, *a priori*, which microbiota species will be effective against a given pathogen. A key challenge is the ecological complexity of the gut. The gut microbiome is a diverse ecological system with many individual species that all have the potential to play a role in colonization resistance. Moreover, these constituent species can also affect each other and interact ecologically in ways that are critical for colonization resistance (12–16). This combination of species diversity and the potential for ecological interactions makes colonization resistance a challenging phenotype to understand (17).

Here we approached the question of mechanisms of colonization resistance from the perspective of the underlying ecological principles. To do this, we studied colonization resistance provided by a range of human gut bacteria, both alone and in combinations. We performed all experiments in parallel using two species of pathogen, which are both on the WHO priority list: *Klebsiella pneumoniae* and *Salmonella enterica* Serovar Typhimurium (18). Both are members of the Enterobacteriaceae found in the human gut microbiome but they have very different lifestyles. *S*. Typhimurium causes acute infection and gastroenteritis (19, 20). By contrast, *K. pneumoniae* is a nosocomial, opportunistic pathogen that rarely causes disease in the gut itself, but gut colonization is a major risk factor for antimicrobial resistance associated infections elsewhere in the body (21).

Despite these differences, we have identified common principles that underlie colonization resistance to both species. Ecological diversity is important for colonization resistance *in vitro* and in gnotobiotic mice. Moreover, we found that colonization resistance is an ecologically complex trait, whereby the ability of one species to provide colonization resistance can depend entirely upon the presence of other species (22). Despite the complexity, we find that these ecological patterns are explained by a simple underlying principle, the collective ability of certain communities to consume nutrients and block pathogen growth. Further, we have shown that this principle offers a way to identify sets of bacterial species that will collectively limit the growth of a particular pathogen.

## Results

### Single species offer little protection in competition with pathogens

**Figure 1:**
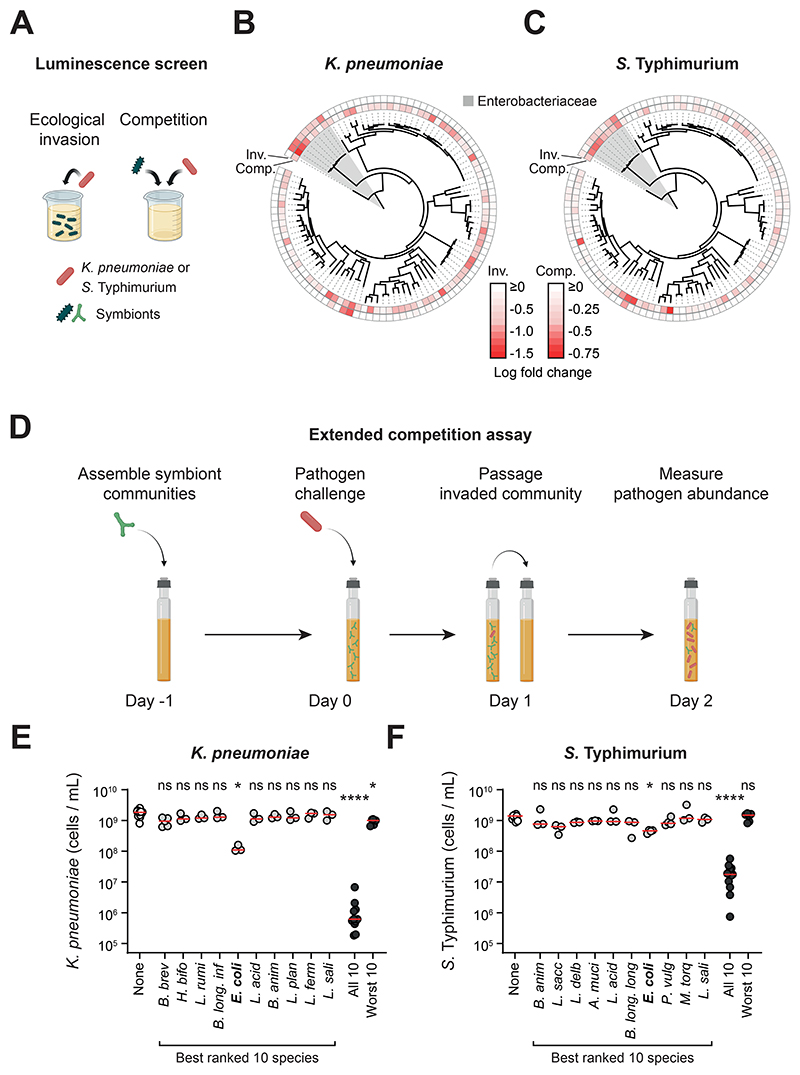
Single strains do not provide robust colonization resistance, but a diverse community can, depending on its composition. **A)** Overview of the luminescence co-culture assays. In the ecological invasion assay, *K. pneumoniae* or *S*. Typhimurium (red) was inoculated in co-culture with individual symbionts (different green symbols are used to represent the diversity of symbiont species screened; 19:1 ratio of symbiont to pathogen). In the competition assay, the symbionts were inoculated at an equal ratio to the pathogen to recapitulate competition between strains once the pathogen is established. In both assays, luminescence produced by the pathogen was used as a proxy for pathogen growth. Created with BioRender.com. **B-C)** Comparison of phylogenetic relatedness between symbionts, and the ability of each symbiont to compete with the pathogen (inv=ecological invasion assay; comp=competition assay). Data for *K. pneumoniae* shown in **(B)** and *S*. Typhimurium shown in **(C)**. The family Enterobacteriaceae, which includes both *K. pneumoniae* and *S*. Typhimurium, is shaded in grey. Luminescence fold change values are presented in Fig. S1. Data presented as the median luminescence log fold change of *N=3-10* independent experiments (biological replicates). Strains with the most negative (most red) values inhibited growth of the pathogen most strongly. **D)** Overview of the extended competition assay. Communities (or individual strains; green) of symbionts are pre-grown in anaerobic rich media before addition of the pathogen (red). The community is passaged after 24h of growth, followed by another 24h of growth before quantification with flow cytometry. Created with BioRender.com. **E-F)** The extended competition assay was performed for each individual species identified in the best ranked 10 species, as well as for combinations of 10 species (both the best- and worst-ranked 10 species; Fig. S1). Individual biological replicates from *N=3-15* independent experiments are shown. Red lines indicate the median. A Kruskal-Wallis test with Dunn’s multiple test correction compares each group to the no-symbionts control (p>0.05=ns; p<0.05=*; p<0.0001=****). Data for *K. pneumoniae* shown in **(E)** and *S*. Typhimurium shown in **(F)**. See Table S1 for species name abbreviations.

Individual members of the microbiota can promote colonization resistance in various contexts (2–8), which suggests that some species are more important for colonization resistance than others. To systematically assess this variability, we screened a diverse set of 100 human gut symbionts (Table S1; also see Methods) for their ability to limit pathogen growth. Competition in the gut occurs both at the point a pathogen enters the gut and when a pathogen becomes established (23, 24). We designed two co-culture assays to reflect these two aspects of competition in the mammalian gut (Fig. 1a). In the first assay (ecological invasion assay), we pre-grew the symbiont alone in standard anaerobic media (modified Gifu anaerobic media; mGAM) buffered to human colonic pH before adding the pathogen. In the second assay (competition assay), we inoculated this media with an equal ratio of symbiont to pathogen, which is designed to capture competition once a pathogen has established itself in the gut.

To assess pathogen growth, we built luminescent strains of *K. pneumoniae* and *S*. Typhimurium and compared luminescence when grown in monoculture and when grown in co-culture with each symbiont. With this assay system, we could rank the strains based on their abilities to limit pathogen growth in both the invasion and competition assays (Fig. 1b-c, S1). From this ranking, we took the top ten best-performing non-pathogenic symbiont species in the screen (Methods; shown in orange in Fig. S1e-f; Table S1) and subjected them to a more stringent test of colonization resistance designed to capture both phases of competition in the gut in one assay (extended competition assay, Fig. 1d). Here, the pathogen is first introduced into a pre-grown culture of a given symbiont strain and then, after 24 hours, the mixture is passaged into fresh media and allowed to grow for 24 hours, whereafter pathogen abundance is assessed via flow cytometry (Fig. 1d). Despite choosing the best-ranked species from the luminescence screen, all symbionts performed poorly under extended competition, with the majority offering no discernible colonization resistance (Fig. 1e-f). The best performer was *Escherichia coli*, a known competitor of *S*. Typhimurium, and also a member of the Enterobacteriaceae, but even here the protection offered was very limited with the pathogens still able to reach 10^8^ – 10^9^ cells/ml.

The outcome of the assay differed greatly when we pooled all ten strains together (Fig. 1e-f). Now, the final abundance of both pathogens was strongly suppressed by over three orders of magnitude for *K. pneumoniae* and about two orders of magnitude for *S*. Typhimurium. By contrast, a community made up of the ten worst-performing strains from the luminescence screen (shown in blue in Fig. S1e-f) provided little or no colonization resistance (Fig. 1e-f). These results, therefore, suggest that strain identity is important for colonization resistance only in the context of a diverse community.

### Ecological diversity and complexity drive colonization resistance *in vitro*

**Figure 2:**
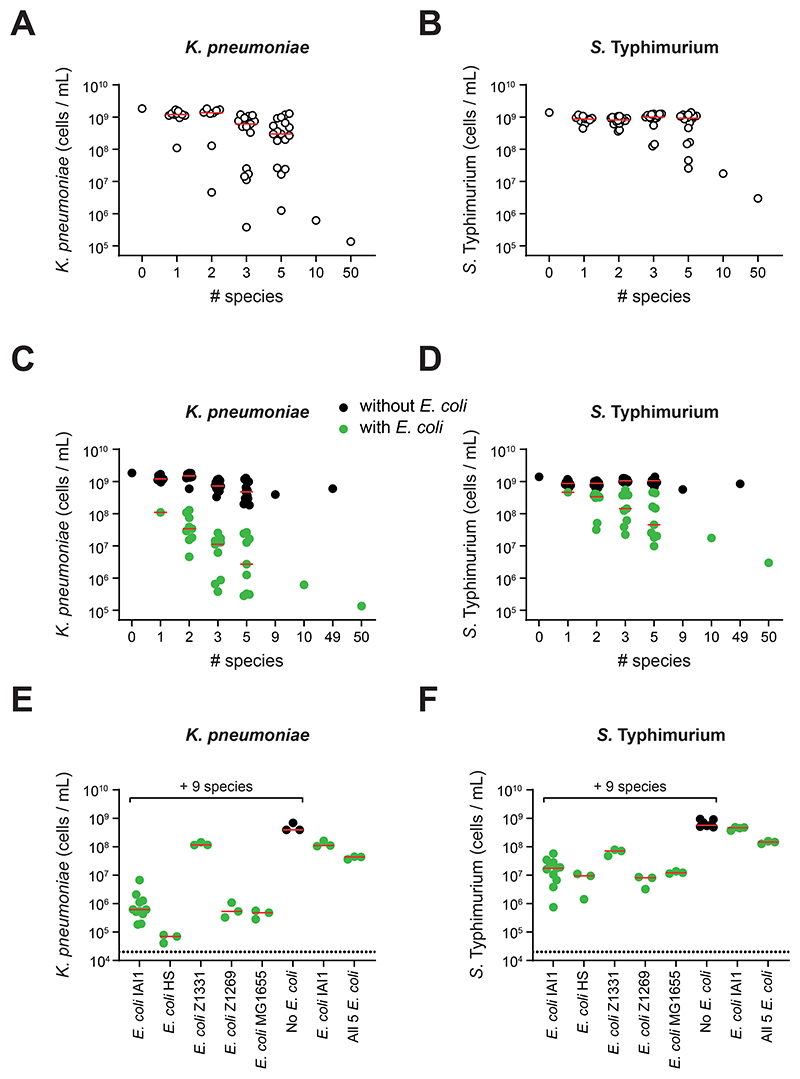
Ecological diversity and key members are needed for efficient colonization resistance *in vitro*. **A-D)** Extended competition assay on communities made up of an increasing number of species. Each data point represents the median pathogen cells/mL value on day 2 of the extended competition for a community (n=3-15 biological replicates from independent experiments for each community; up to 17 communities for each group). Communities with size <= 10 species were randomly selected from the 10 best ranked species for each pathogen. Community identities are shown in Table S4-5. Data for *K. pneumoniae* shown in **(A)** and **(C)**, and for *S*. Typhimurium shown in **(B)** and **(D)**. Red lines indicate the median value of communities at a given diversity level. In **C-D)**, data from **A-B)** are replotted along with additional communities that always contained *E. coli* but were otherwise randomly selected. Communities without *E. coli* are depicted in black; communities with *E. coli* in green. Separate red median lines shown for communities with and without *E. coli*. A linear regression is performed on log-log transformed data in Fig. S2a-b, which shows that the association between diversity and colonization observed is statistically significant, and that this effect is greater for communities with *E. coli* than those without (F tests, p≤0.0001). **E-F)** Extended competition assay testing *E. coli* strains substituted into the best ranked 10 species community. Data for *K. pneumoniae* shown **(E)** and *S*. Typhimurium in **(F)**. Red lines indicate median values. Each data point represents a biological replicate each from independent experiments (*N=3-11*).

Our results indicated that microbiota diversity is important for colonization resistance. This finding fits well with the general idea that microbial diversity is beneficial for microbiome functioning, whereas a loss of diversity, or dysbiosis, can be associated with poor health and disease (25–27). While the potential benefits of diversity are clear, cause and effect can be confounded in observational studies (28). To systematically test the role of diversity in colonization resistance, we randomly selected communities of increasing diversity from the best ranked 10 strains and competed them against the pathogens in the extended competition assay. To further evaluate the importance of diversity, we also assembled a community of 50 non-pathogenic symbiont species from the strains in our initial luminescence screen (see Methods). These data indicated a relationship between diversity and colonization resistance. However, we also saw a large variation in colonization resistance across the communities that differed in their composition of two, three and five species. Visual inspection of the data (Fig. 2c-d) suggested that a large component of this variability was driven by the composition of the communities.

One species that appeared to be important for outcomes was *E. coli*. To explore this finding, we randomly selected additional *E. coli*-containing communities and again evaluated colonization resistance (Fig. 2c-d). We also performed drop-out experiments, where we made up the 10 and 50 species communities without *E. coli* (Fig. 2c-d). These data revealed a strong and clear monotonic increase in colonization resistance as species diversity increases (Fig. 2c-d, **green circles**), but this relationship is much weaker or disappears entirely in the absence of *E. coli* (Fig. S2a-b). In ecological terms, these data show that colonization resistance rests upon a strong higher-order effect involving other community members and *E. coli* (22). By higher-order effects here, we mean cases where the effect of one species on another is changed by the presence of a third-party species in a community (22). That is, while *E. coli* alone or the rest of the community alone each have little impact on pathogen growth, together they have a strong effect on pathogen growth. Such higher-order effects are considered important in ecology as they imply context dependence, which can make a system difficult to understand and predict (22, 29–32). Another way to illustrate the effect of diversity on colonization resistance is to compare our data to a simple null model. Consider, for example, a model where each additional species proportionally improves colonization resistance to the pathogen. Specifically, we can compare our experimental data for *E. coli* containing communities to a null model where pathogen abundance scales according to 1/*n*, where *n* is the number of species present. This analysis shows that the deviation from such a null model increases as diversity increases, where colonization resistance is again greater than expected for diverse communities (Fig. S3).

We also asked whether the role of *E. coli* within communities was a strain-specific effect. We replaced *E. coli* strain IAI1, identified in our screen, by each of four other *E. coli* strains historically isolated from the human gut (33–35). The effect of *E. coli* was similar when *E. coli* IAI1 was substituted by most *E. coli* strains (Fig. 2e-f), which indicates that the higher-order effect involving *E. coli* is a general property of closely-related strains.

Further inspection of the data pointed to other species that were important for colonization resistance in diverse communities. In *E. coli* containing communities, the presence of *Bifidobacterium breve* appeared to be important in excluding *K. pneumoniae* (Fig. S4a), and *Lacrimispora saccharolyticum* and *Phocaeicola vulgatus* in the exclusion of *S*. Typhimurium (Fig. S4b). We confirmed these patterns through a series of systematic drop-out experiments (Fig. S4c-d). However, it was still possible to achieve equivalent colonization resistance in more diverse communities that lack these species (Fig. S4c-d), which again points to the underlying benefits of a diverse microbiota.

### Ecological diversity and complexity also drive colonization resistance *in vivo*

**Figure 3:**
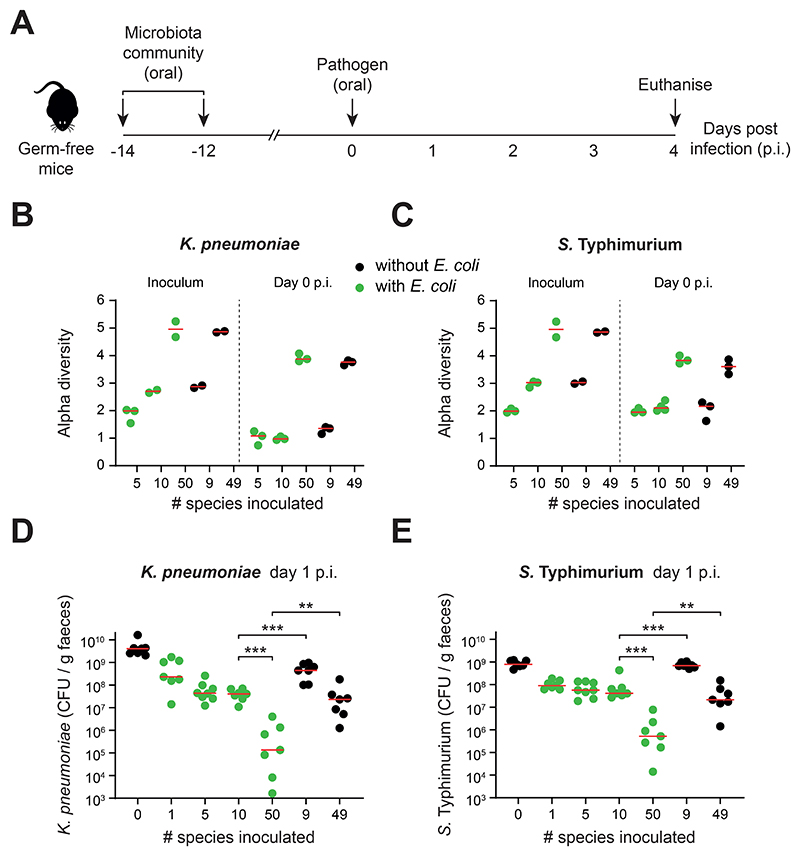
Ecological diversity and key members are needed for efficient colonization resistance *in vivo*. **A)** Overview of gnotobiotic mouse experiments. Symbiont communities (or *E. coli* alone) were given to germ-free mice by oral gavage twice (two days apart). 12 days later, the mice were challenged with *K. pneumoniae* or *S*. Typhimurium by oral gavage. Feces were collected from mice daily before being euthanised on day 4 post infection (p.i.). **B-C)** Alpha diversity measured by Shannon index of symbiont communities. Metagenomic sequencing was performed on the inoculum and fecal samples at day 0 (when the pathogen is introduced) and used to calculate diversity. Data for *K. pneumoniae* shown in (**B**) and *S*. Typhimurium in (**C**). Biological replicates from a representative mouse from each cage are shown (*N=2-4*; at least two independent experiments). **D-E)** Pathogen abundances in the feces of gnotobiotic mice colonized with communities of increasing diversity (mice containing communities with *E. coli* shown in green; mice containing communities without *E. coli* shown in black; *N=7-8* biological replicates of mice per group in cages of 2-3 mice; 2-3 independent experiments). Red lines indicate the medians. Two-tailed Mann-Whitney tests are used to compare the indicated groups (p<0.01=**; p<0.001=***). Data for *K. pneumoniae* shown in **(D)** and *S*. Typhimurium shown in **(E)**. Metagenomic analysis of strain diversity and relative abundance is shown in Fig. S5. Pathogen abundance data from days 1-4 p.i. is shown in Fig. S6. Community compositions are shown in Table S6.

To validate our *in vitro* methods, we tested the ability of symbiont communities to resist pathogen colonization in gnotobiotic mice (Fig. 3a). Germ-free mice were colonized with symbiont communities differing in diversity, and the presence or absence of *E. coli*. Successful colonization by *S*. Typhimurium causes an acute infection and massive gut inflammation, which is a major confounding effect for studying the effects of community composition on pathogen growth. Animals with a less protective microbiota can rapidly succumb to the infection, such that one cannot follow ecological dynamics over time in a comparable way across treatments. We, therefore, chose to use an avirulent variant of *S*. Typhimurium to eliminate the effect of gut inflammation on pathogen and host, where one can use pathogen abundance as a measure of disease risk (19, 36, 37). We introduced communities across the same range of diversities as before but, in contrast to *in vitro* assays, not all symbiont species will reliably colonize germ-free mice (38). Therefore, we used metagenomic sequencing to confirm that introducing a higher diversity community to the mice did indeed result in a higher diversity of strains colonizing the gut as measured by two metrics of alpha diversity, and to identify the relative abundance of all members (Fig. 3b-c, S5).

These experiments revealed that, as observed *in vitro*, microbiome diversity is negatively correlated with pathogen abundance in feces for both pathogens (compare 10 vs 50-member communities; Fig. 3d-e; Fig. S6). Moreover, drop-out experiments revealed again the importance of the combination of *E. coli* and other community members for colonization resistance (Fig. 3d-e). We also observe that higher diversities are needed for efficient colonization resistance in the mammalian gut than in our *in vitro* assays, which is likely to be explained by the higher degree of environmental and spatial heterogeneity in the gut compared to a test tube. Nevertheless, the key patterns remain the same between the gnotobiotic mouse experiments and our *in vitro* assays. Both ecological diversity and higher-order interactions are important for colonization resistance to both pathogens. As before, we saw a strong deviation from a simple null model of ecological competition at high levels of diversity (Fig. S3). In addition to showing the generality of these patterns, this fit between the *in vitro* and *in vivo* methods validates our extended competition assay as an approach to interrogate the ecology of colonization resistance.

### A simple principle explains the roles of diversity and complexity in colonization resistance

The discovery of such higher-order effects in colonization resistance indicates that colonization resistance is an ecologically complex trait (22), which can be challenging to work with owing to high levels of context dependence (17, 22, 29, 30). Nevertheless, we sought to understand the mechanisms underpinning colonization resistance by returning to our *in vitro* data gathered from large numbers of different communities. The genomic data enabled us to assess functional similarity between symbiont communities and pathogens from overlap in protein compositions. Specifically, we calculated the percentage of all protein families carried by a pathogen that were also present in each community investigated (see Methods). We reasoned that this measure of functional similarity may map to niche overlap and, therefore, the strength of ecological competition between symbionts and pathogens. We first confirmed that the number of encoded protein families covered by our experimental communities increases proportionally with the number of added strains (Fig. S7). Permutation analyses also confirmed that the randomly selected communities we have studied experimentally are a good representation of all possible communities that we could have studied (Fig. S8).

**Figure 4:**
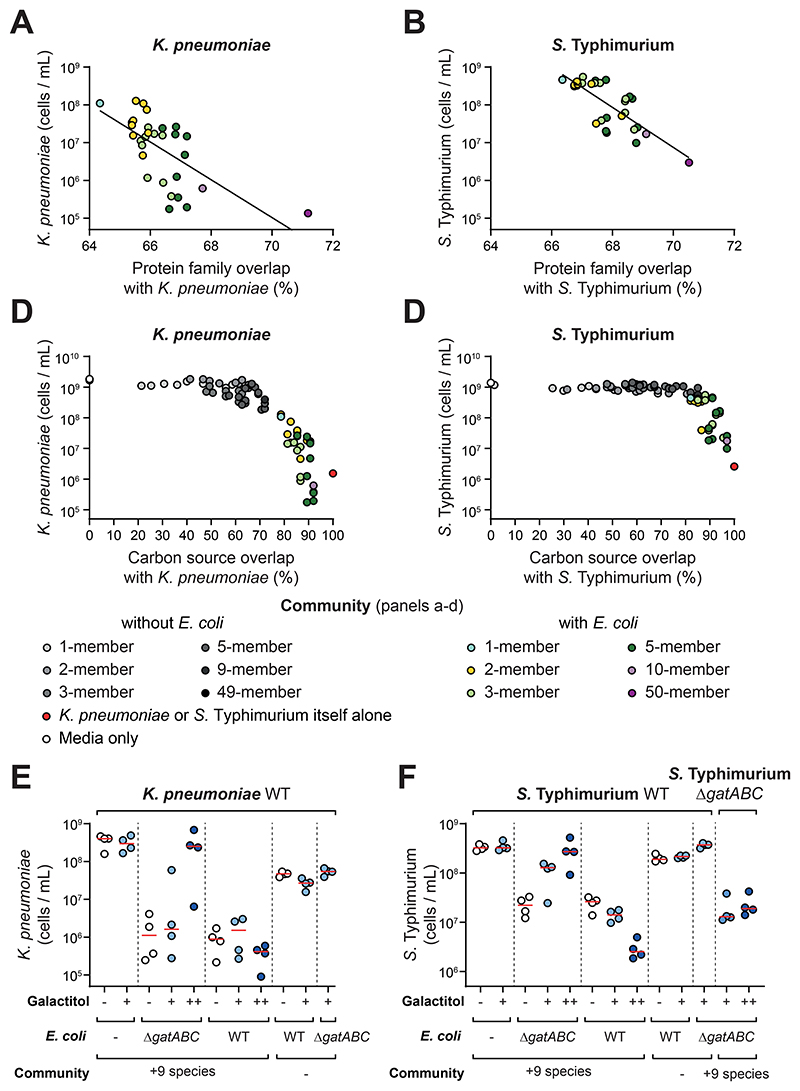
Nutrient overlap can explain the role of ecological diversity and the effect of *E. coli* in colonization resistance. **A-B)** Protein family overlap is compared to the median pathogen abundance values for each community containing *E. coli* from Fig. 2C-D. Diversity is visualised by a color gradient. Data for *K. pneumoniae* shown in **(A)** and *Salmonella* shown in **(B)**. A line of best fit is shown from a linear regression on log transformed data: R^2^ = 0.4255 for *K. pneumoniae*; R^2^ = 0.603 for *S*. Typhimurium; both slopes are significantly different from 0 using an F-test (p<0.0001). Data for communities without *E. coli* is presented in Fig. S9E-F. **C-D**. Overlap in carbon source utilisation plotted against the median pathogen abundance measurements from experimental communities in Fig. 2C-D. Community carbon source overlap is calculated using an additive approach from carbon source overlap of individual strains by measurement on AN Biolog Microplates (Fig. S10). Diversity is visualised by a gradient of color (for *E. coli-*containing communities) or greyscale (for communities without *E. coli*). A control with the isogenic pathogen itself (100% overlap) is plotted in red. Data for *K. pneumoniae* in **(C)** and *S*. Typhimurium shown in **(D). E-F)** A private nutrient, galactitol, that could only be used by the WT *E. coli* strain and the pathogens but not by the other symbionts nor an *E. coli* Δ*gatABC* mutant, was supplemented to the media and the extended competition assay performed as before. In all treatments, pathogen abundance was measured by flow cytometry after 48h of growth post-passage instead of the usual 24h. This change did not influence the control experiments without galactitol, but proved informative because we found the growth impacts of galactitol were relatively slow. Results for *K. pneumoniae* shown in **(E)** and *S*. Typhimurium in **(F)**. *N=3-4* biological replicates from independent experiments per treatment. Horizontal red lines show the median of the replicates. Light blue circles show results with 0.1% galactitol supplementation (+ symbol), dark blue circles show results with 1% galactitol supplementation (++ symbol). White circles (control) show results with no nutrient supplementation (- symbol). 9 species refers to the 9 additional species in the 10 best-performing species for each respective pathogen (- symbol refers to when *E. coli* is added alone). In (**D**), a Δ*gatABC* mutant of *S*. Typhimurium was used in addition to the WT pathogen to verify the dependency of colonization on a private nutrient.

The potential importance of protein family overlap was already clear from the effects of *E. coli* in our experimental data (Figs. 2-3). *E. coli* is in the same family of bacteria as *K. pneumoniae* and *S*. Typhimurium and can be seen to contribute greatly to the overlap in the protein families carried by a given community and either of the pathogens in our experimental communities (Fig. S9a-d). However, by taking only the communities that contain *E. coli* to control for this effect, we also see a strong correlation between a community’s protein family overlap with the pathogen and its colonization resistance in our *in vitro* assays (Fig. 4a-b; Fig. S9). In other words, if the symbiont strain or community encoded many of the same (or similar) proteins as the pathogen, it provides better colonization resistance. The same analysis for communities that lack *E. coli* is not informative as colonization resistance is consistently so low across all communities (Fig. 2).

Altogether, our genomic analysis suggests that communities that overlap highly with the pathogens in encoded functions provide the best colonization resistance. These analyses support our hypothesis that niche overlap is important for our observed ecological patterns in colonization resistance. One of the key drivers of niche overlap is resource competition (39, 40), which is a known contributor to colonization resistance to *K. pneumoniae* and *S*. Typhimurium (12, 13). We, therefore, explored the role of nutrient competition by generating metabolic profiles for the two sets of 10 key symbiont species identified in the original screen against each pathogen (Fig. 1) using AN Biolog MicroPlates that profile the metabolic activity of each strain on 95 carbon sources (Fig. S10). Note that to cover the two sets of 10 species, we only profiled 16 strains in practice because there were some parallels between the two sets of top-ranked strains in the luminescence screen. We first established that there was a strong positive association between the protein family (genomic) and metabolic (Biolog) overlap of communities with the pathogens (Fig. S11). We then assessed the ability of metabolic overlap to predict colonization resistance (Fig. 4c-d). Colonization resistance was only observed once communities shared sufficiently high overlap in their carbon source utilisation profile with a pathogen. Moreover, communities with the greatest metabolic overlap with a pathogen provided the greatest colonization resistance. An important observation from these data is that it is not diversity *per se* that predicts colonization resistance, it is the overlap between the pathogen and the communities. This pattern is made clear by the observation that communities of different diversities, but the same overlap, appear proximally in the plots (neighbouring points of different color in Fig. 4c-d).

Overall, our data point to the importance of nutrient competition, and specifically nutrient use overlap between a community and a pathogen, as an explanation for the patterns we observe in colonization resistance. To further support this conclusion, we performed experiments where the pathogens were grown in cell-free (spent) media collected from different communities, which excluded cell-cell contact mechanisms as explanations for colonization resistance. Growing the pathogen in the spent media of *E. coli* and the 10 species communities recapitulated the patterns seen in the competition experiments, consistent with the effect of nutrient competition (Fig. S12). As a final test, we sought a nutrient that can be used by the pathogens only and used it to perform nutrient supplementation experiments (Fig. 4e-f). We identified galactitol from the Biolog plates (Fig. S10). The pathogens can use this sugar alcohol but it has the desirable property that it cannot be used by any of the symbionts in our focal 10-species communities, except for *E. coli*. We engineered a strain of *E. coli* that lacks the transporter for cell import (*E. coli gatABC* deletion mutant). By adding in galactitol to our standard media, we found that colonization resistance in a diverse community is lost if the pathogens can use the nutrient but *E. coli* cannot (Fig. 4e-f). However, colonization resistance is restored when *E. coli* can use the nutrient. Further, if a pathogen is engineered so that it cannot use galactitol (*S. Typhimurium gatABC* deletion mutant), colonization resistance is restored. These outcomes are exactly as expected if nutrient competition is the cause of colonization resistance.

Our data show that the ability of a microbiota community to consume nutrients required by a pathogen for growth underlies the colonization resistance we observe. Importantly, the nutrient blocking effect is a property of the whole community rather than any one species alone. That colonization resistance is a community-level trait explains the importance of the ecological diversity, and complexity (22) we observed in our experiments. Despite considerable genomic and metabolic overlap with the pathogens, a species like *E. coli* cannot alone block enough nutrients to provide colonization resistance. It is only in combination with other species, that *E. coli* becomes effective at limiting pathogen growth.

### Nutrient blocking can identify protective communities

Our experiments indicate that colonization resistance is an ecologically complex trait, but that this complexity can be understood and predicted via a simple underlying principle. As an additional test of these findings, we used the nutrient blocking to predict community compositions that provide colonization resistance to a bacterial strain that was not present in our initial experiments. For this test, we chose an antimicrobial resistant (AMR) clinical *E. coli* strain, which was isolated from the urine of a patient. AMR *E. coli* strains are a major current target for alternatives to antibiotics because members of this species have recently been found to be responsible for the most AMR-associated deaths of any bacterial species (41).

**Figure 5:**
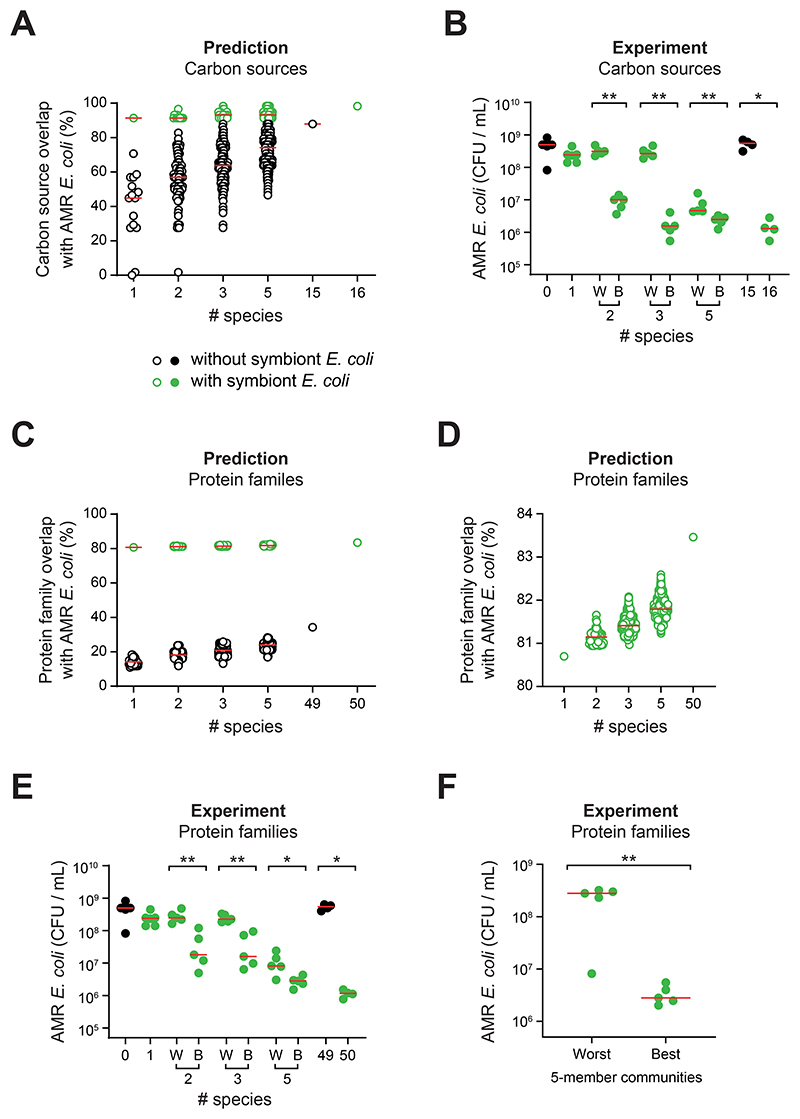
Nutrient blocking predicts community colonization resistance. **A)** *In silico* prediction of carbon source overlap with the AMR *E. coli* strain for all possible combinations of symbiont communities at the indicated diversity levels. Each circle represents a different community. Communities containing the symbiont *E. coli* IAI1 are shown in green and communities without *E. coli* IAI1 are shown in black (predictions as hollow circles; experimental data as solid circles). Predicted carbon source overlap calculated using an additive approach from carbon source use of individual strains measured using AN Biolog MicroPlates (Fig. S10, S13). **B)** Experimental test of *in silico* predictions in **(A)**. The two *E. coli* IAI1-containing communities predicted to have the best (B) and worst (W) carbon source overlap were picked at each diversity level and competed against AMR *E. coli* in the extended competition assay. A two-tailed Mann-Whitney U test was performed on community pairs (p>0.05=ns; p<0.05=*; p<0.01=**) at the 2-, 3- and 5-strain diversity levels. Red horizontal bars depict the median of each community tested. *N=4-5* biological replicates from independent experiments for each community. **C-D)** *In silico* prediction of protein family overlap with the AMR *E. coli* strain for a random subset (n=59,043) of all possible symbiont communities at diversity levels 2-, 3-, and 5-strains, as well as all individual strains and the 49- and 50-species communities. Each circle represents a different community. Communities are selected from the strains comprising the 50-member community. Communities containing *E. coli* IAI1 are shown in green and communities without *E. coli* IAI1 are shown in black. **D)** Only the *E. coli*-containing communities are plotted to better visualise variation in protein family overlap. **E)** Experimental test of *in silico* predictions based on protein cluster overlap in **(C-D)**. The two *E. coli* IAI1-containing communities predicted to have the best (B) and worst (W) protein family overlap were picked at each diversity level (randomly selected, for cases where there were multiple communities with the same overlap), and competed against AMR *E. coli* in the extended competition assay. Red horizontal bars depict the median of each community tested. N=5 biological replicates from independent experiments for each community. A two-tailed Mann-Whitney U test was performed on community pairs (p>0.05=ns; p<0.05=*; p<0.01=**) at the 2-, 3- and 5-strain diversity levels. **F)** Experimental test of the predicted 5 best and 5 worst communities at the 5-strain diversity level, based on protein family overlap with AMR *E. coli*. Each symbol represents the median of N=5 biological replicates from independent experiments per community. Red horizontal bars depict the median of the best and the worst predicted communities. A two-tailed Mann-Whitney U test was performed (p<0.01=**). Community identities for (**B, E-F**) are shown in Table S7.

We first analysed the AMR *E. coli* isolate on AN Biolog MicroPlates to assess its carbon source utilisation and compared this to the top ranked strains from our initial luminescence screen (Fig. 1). We reasoned that these top-ranked strains were a good place to start as *E. coli* is also a member of the Enterobacteriaceae, like the two pathogens that were used to select the top-ranked strains. As expected, the AMR *E. coli* had the greatest protein overlap with the symbiont *E. coli* in our 16 strains but, importantly, additional strains were predicted to be required to block nutrient availability based on the overlap needed to suppress the two pathogens (Fig. S13). We next used the Biolog data to computationally assemble all possible communities of one, two, three and five species from the 16 strains and calculated their resource utilisation overlap with the AMR *E. coli* (Fig. 5a). Again, in line with our findings, diversity improved the median resource utilisation overlap, but this depended strongly on the presence of the symbiont *E. coli*.

The simplest test of the importance of nutrient blocking is to remove the symbiont *E. coli* from a community and test the impact. Doing this for the community of all 16 strains confirmed the importance of *E. coli* for colonization resistance (Fig. 5b). However, we also tested our ideas on communities that contain *E. coli*. In these experiments we identified communities predicted to have the highest and lowest overlap with the target strain at each diversity level, where communities were randomly chosen if there were ties in rank. We then used our extended competition assay (Fig. 1d) to test the ability of the AMR *E. coli* to invade the communities. As predicted by nutrient blocking, this revealed that increasing diversity leads to increased colonization resistance and, critically, for each diversity level, the community predicted to resist the AMR *E. coli* consistently performed better in colonization resistance than the community predicted to do poorly (Fig. 5b). This result was clearest for the two and three species communities. For the five species community, the best performing community was only marginally better than the worst. We reasoned that this was because, in these experiments, we are limited to choosing from 16 strains that were preselected for being relatively good competitors to Enterobacteriaceae (Fig. 1b-c, S1).

To test the nutrient blocking principle more robustly we selected from a wider range of possible strains from our set of 50 strains that we used in our *in vitro* and *in vivo* experiments (Fig. 2-3). Most of these strains had not been characterised for their functioning in community-level colonization resistance, other than in the 50 species treatment. We also used this set of experiments to test the power of the nutrient blocking principle to predict colonization resistance based upon genomic data alone. Rather than using the Biolog phenotypic assay, therefore, we returned to our measure of protein family overlap, which calculated the overlap in all protein types between an invading strain and different communities. To do this we only had to sequence the AMR *E. coli* clinical isolate because all 50 other strains were sequenced. Using the same approach as for the Biolog predictions, we then assembled communities *in silico* that all contained the symbiont *E. coli* strain and, in each case, calculated their protein family overlap with the AMR *E. coli* (Fig. 5c-d). As before, we chose communities with the lowest and highest overlap to the AMR *E. coli* across a range of diversities (randomly choosing communities if there were ties in rank) and experimentally assessed colonization resistance using the extended competition assay.

We again see the importance of community diversity in these experiments. Moreover, despite using only genomic information and a much larger set of possible communities, we observed improvement in colonization resistance from the worst to best communities at each diversity level (Fig. 5e). Finally, we evaluated our ability to select highly and poorly performing communities by assessing colonization resistance in additional five-species communities. Notably, at the five-species level, more than 200,000 communities with *E. coli* can be assembled from 50 strains. We used our algorithm to sample approximately 50,000 and, from these, we identified four additional community compositions predicted to perform well and four predicted to perform poorly (to give five of each class). In line with our predictions, the communities predicted to be colonization resistant showed a median 100-fold reduction in the abundance of the AMR *E. coli* compared to those predicted to be permissive (Fig. 5f).

## Discussion

A key benefit of the microbiome is its ability to reduce the probability of infection via colonization resistance (1, 2, 10, 42). Here we have used an ecological approach to understand the principles of colonization resistance in the gut microbiome. By screening a collection of human gut symbionts, we found that individual strains were unable to provide effective resistance to pathogens (Fig. 1), but that colonization resistance increases monotonically with ecological diversity (Fig. 2-3). Our work, therefore, supports the general hypothesis that a more diverse microbiome can carry health benefits (28, 43–45). While much discussed, evidence for this hypothesis is typically based upon correlations between microbiome diversity and health outcomes (28, 45, 46). Here, we provide experimental evidence that microbiome diversity can provide health benefits via an increased ability to protect against pathogens. Moreover, we explained this pattern in terms of the importance of the overlap between the nutrient requirements of an invading pathogen and the resident community (Fig. 4).

We found that certain combinations of species display much greater colonization resistance together than when alone. These non-additive effects mean that colonization resistance is formally a complex ecological trait in the canon of ecology (22). Such effects are often assumed to imply a complex network of interactions between species where, for example, one symbiont species affects a second symbiont species and changes the way this second species interacts with a pathogen. However, consideration of nutrient competition and particularly the level of overlap between a pathogen and community revealed that much simpler processes explain the complexity we see. One species alone is not sufficient to strongly impact pathogen growth, but rather a combination of species is required to block nutrient access. Interestingly, the combinations of species that make colonization-resistant communities are often very phylogenetically diverse. Generating resistant communities does not rest upon simply finding closely related species to a given pathogen, in our case against Enterobacteriaceae members. Instead, a mixture of gram-positive and gram-negative species is often what performs best (Fig. 5f, Table S7). While both of our pathogens are members of the same family, they have different life histories (19–21). *S*. Typhimurium is a specialist gut pathogen while *K. pneumoniae* is an opportunistic pathogen that typically causes no pathology in the gut itself, instead causing infections in other parts of the body. Consistent with this, we find it is consistently easier to generate colonization resistance to *K. pneumoniae* than *S*. Typhimurium. Nevertheless, the importance of ecological diversity and complexity is observed for both pathogens. We anticipate that the importance of ecological diversity and the principle of nutrient blocking will apply generally, given the widespread evidence that nutrient competition is important for diverse species in the microbiome, including many other pathogens (9, 47–55).

By assembling a wide range of communities of defined compositions, we have been able to establish links between ecological diversity, complexity and nutrient competition in colonization resistance. However, a limitation of this approach is that we have focused on relatively low diversity communities (up to 50 species). A question for the future is whether our findings will hold for the higher levels of diversity that can naturally occur in the human microbiome. Consistent with our findings, colonization risk with species like *K. pneumoniae* is increased after antibiotic treatment that can lower species diversity in the microbiome (56). Nutrient competition is central to the patterns that we have described here. Colonization resistance can develop via additional mechanisms, which include toxin-mediated bacterial competition and effects via the host immune system (1, 10). Our work does not exclude the potential importance of these additional mechanisms such as direct killing of invading strains by members of the community, which can act in parallel to nutrient competition (12, 13, 15, 39). Moreover, our predictions of resistant and permissive communities are not perfect (e.g., high overlap communities in Fig. 4c; the outlier in Fig. 5f). However, it is notable that these deviations are in the direction of a community being more resistant than expected. This suggests that predictions based upon nutrient blocking may often be conservative with errors resulting in communities performing better than expected whenever other mechanisms of colonization resistance are at play.

Our work shows that colonization resistance is not the property of single microbiome species but instead the collective property of multiple species. Specifically, we find that the effect of a given symbiont species on a pathogen can be strongly dependent on whether other symbiont species are also present to consume nutrients that the pathogen needs. This finding suggests that one can use the idea of nutrient blocking to identify sets of microbiome species that will limit the growth of a target strain. As a proof-of-principle, we tested this idea for an AMR *E. coli* strain, which revealed that species sets can be successfully identified that collectively suppress an incoming strain (Fig. 5). Importantly, we find that this can be done without specific information on available nutrients or the metabolism of the species under study. Instead, a measure of genomic overlap can be used as a proxy for niche overlap to assemble communities that perform nutrient blocking (Fig. 5c-f). The human microbiome is dauntingly complex and has great potential for context-dependent effects. However, we found that microbiome complexity can arise via simple underlying principles, which gives promise to the goal of rationally designing microbiomes for better health.

## Materials and methods

### Bacterial strains and plasmids

A full list of bacterial strains used in this study is provided in Table S1. The plasmids used in this study are listed in Table S2. *Klebsiella pneumoniae* subsp. *pneumoniae* purchased from DSMZ (stock number 30104) was used in all experiments containing *K. pneumoniae. Salmonella enterica* serovar Typhimurium (strain SL1344 (57)) was used in all experiments containing *S*. Typhimurium. The 100 strains were chosen for being abundant and/or important members of the human gut microbiota that cover the key phylogenetic groups found in the human gut microbiota. In addition, all are type strains, have had their genome sequenced (which we rely on for the protein family analysis), and are known to be culturable in nutrient-rich, anaerobic medium (mGAM) (58, 59), so were conducive to experimental work. Strains were kindly provided by Nassos Typas (EMBL Heidelberg, Germany), or were ordered from the German Collection of Microorganisms, DSMZ or the American Type Culture Collection (ATCC; Staph epidermidis). The *Lactobacillus plantarum* strain was provided by the Department of Food and Nutritional Sciences, University of Reading. *Klebsiella pneumoniae* ATCC 700721 was provided by the Modernising Medical Microbiology research group, Nuffield Department of Medicine, University of Oxford. *Escherichia coli* strains Z1331 and Z1269 were isolated from the feces of two healthy human donors in Switzerland (33). The ampicillin-resistant AMR *E. coli* strain is a urine clinical isolate provided by the Pathogen Bank at Nottingham University Hospitals NHS Trust.

### Bacterial growth conditions

For engineering of *E. coli, K. pneumoniae*, or *S*. Typhimurium, strains were grown aerobically in Lysogeny broth (LB; Fisher Scientific) with the appropriate antibiotics (Table S1) at 37°C, shaking at 220 rpm. All symbionts were cultured under anaerobic conditions (5% H_2_, 5% CO_2_, 90% N_2_, <20ppm O_2_) in modified Gifu Anaerobic Medium (mGAM; Nissui Pharmaceuticals) broth buffered to pH 6.2 with 100mM 2-(N-morpholino)ethanesulfonic acid (MES; Sigma-Aldrich). The redox indicator dye Resazurin (100μg/L media; Sigma-Aldrich) was added as a quality control check for the presence of oxygen (turns red when conditions are not sufficiently anaerobic). To prepare glycerol stocks, individual strains were first streaked onto mGAM agar (Nissui Pharmaceuticals) and grown under anaerobic conditions (if possible, as not all strains can grow as single colonies on agar-based media). The identity of each strain was confirmed using 16S rRNA sequencing (Sanger sequencing; Source Biosciences) using the primers oOPC-953 and oOPC-954 for most species, or g-Bifid-F and g-Bifid-R for Bifidobacteria (Table S3). Single colonies were then inoculated in mGAM broth and stored at -70 degrees Celsius in mGAM with a final concentration of 25% glycerol.

### Genetic engineering of bacterial strains

Luminescence and fluorescence plasmids were transformed into *K. pneumoniae* and *S*. Typhimurium using electroporation. Briefly, 5mL overnight culture was washed 3x with cold Milli-Q water, before being concentrated in 500μl cold Milli-Q water. 2μl of plasmid was mixed with 100μl of concentrated cells and electroporated (1.8kV; 0.1cm gap cuvettes) before recovery in 1ml pre-warmed LB (1h at 37°C, shaking at 220rpm) and plating on LB with the appropriate antibiotics.

Gene deletions were generated as described in (60). Briefly, 700 base pairs upstream and downstream of region to be deleted were PCR amplified (Phusion^®^, NEB) and inserted into the suicide vector pFOK, which was linearised using the restriction enzymes BamHI and EcoRI, using the NEBuilder^®^ HiFi DNA Assembly (NEB). The plasmid was introduced into a diaminopimelic acid auxotroph E. coli strain (JKe201). After 6h of mating between the plasmid-containing donor *E. coli* strain and the recipient *E. coli, S*. Typhimurium or *K. pneumoniae* strain, trans-conjugants were selected on LB plates containing 50μg/mL kanamycin. Counter-selection was performed on no-salt LB plates supplemented with 0.5μg/mL of anhydrous tetracycline and 20% of sucrose at 30ºC. Mutants were screened by colony PCR and the sequence was verified using Sanger sequencing (Eurofins). Primers used for genetic engineering in this study are listed in Table S3.

### Luminescence screen

*K. pneumoniae* (DSM 30104) and *S*. Typhimurium (SL1344) carrying a low copy number plasmid with *P*_nptII_ promoter-driven expression of *lux*CDBAE-*frp* (pRSJ-p_nptII_::ilux; also known as the improved lux operon plasmid (61)) were used for the luminescence screen. Symbiont strains were tested in pairwise co-culture with the pathogens. All strains tested were first grown in monoculture anaerobically in static mGAM broth buffered to pH 6.2 with 100mM MES at 37ºC. *K. pneumoniae* WT and *S*. Typhimurium WT carrying the luminescence plasmid pRSJ-p_nptII_::ilux were separately incubated in the same media overnight.

#### Competition assay

once all strains had reached stationary phase (12-72 hours of growth depending on the species), 20μL of each symbiont strain was dispensed in technical triplicates in a 96-well plate containing 160μL of mGAM broth. 20μL of *K. pneumoniae* pRSJ-p_nptII_::ilux overnight culture or S. Typhimurium pRSJ-p_nptII_::ilux overnight culture was then added to each well.

#### Ecological invasion assay

180μL of each symbiont strain was dispensed in technical triplicates in a 96-well plate and 20μL *K. pneumoniae* pRSJ-p_nptII_::ilux overnight culture or *S*. Typhimurium pRSJ-p_nptII_::ilux overnight culture was added to each well.

96-well plates from both assays were incubated anaerobically for 6 hours at 37ºC. Before luminescence measurement, 96-well plates were brought out of the anaerobic chamber and exposed to O_2_ for 10 minutes because, critically, O_2_ is needed for light production catalysed by the enzyme luciferase. Luminescence at 515-575nm and OD-600nm were measured using a CLARIOstar Plus spectrometer (BMG Labtech). Each data point (Fig. S1) represents the median of at least 3 independent biological replicates (that is, using different overnight cultures of the same strain in different 96-well plates).

### Selection of the top and bottom ten symbiont strains from the screen

For each of the ecological invasion and competition assays, symbiont strains were ordered from best- to worst-performing. Symbiont strains were given two rankings: one from the competition assay and one from the invasion assay. The two rankings were summed and ordered from lowest sum (best overall performing) to highest sum (worst overall performing). From these ranks, the top ten and bottom ten strains were chosen. In order to focus on the ability of non-pathogenic strains to limit colonization by pathogens, we decided to focus for the community experiments on strains that have a category 1 safety level. Many common gut bacteria cause occasional infections and therefore categorised as opportunistic pathogens with a category 2 safety level, which can blur the line between being protective and causing disease. Removing these strains took us from 100 to 54 strains. From these, we selected 50 species as our core set, removing some strains that were duplicates of the same species.

### Construction of phylogenomic tree

The phylogenomic tree of strains in the luminescence screen (n=100; Fig. 1b-c; Table S1) was inferred using default settings based on 67 single-copy core genes using anvio v7.1 (62) and plotted using iTOL v6.7.5 (63).

### Extended competition assay

All culturing was performed in a shaking incubator at 225rpm and 37ºC, and under anaerobic conditions. Symbiont strains were grown to stationary phase (12-72 hours, depending on the species), in Hungate tubes containing 5mL mGAM broth and 100mM MES and buffered to pH 6.2. *K. pneumoniae* and *S*. Typhimurium carrying the fluorescence plasmid pBC11 (YPet) (64) were incubated overnight, with the appropriate antibiotics (Table S1).

Once in stationary phase, monocultures of symbionts were passaged (100μL culture into a new 5mL tube of media) and grown for ~17 hours. Communities were assembled under anaerobic conditions into new 5mL Hungate tubes (see section “community preparation”) and grown for 24 hours. Once grown, communities were invaded with 100μl of the pathogen (10^6^ cells/mL final concentration) in each Hungate tube containing 5mL culture. Samples were taken immediately after addition of the pathogen and prepared for measurement using flow cytometry (day 0). After 24 hours growth, the invaded communities were sampled for flow cytometry (day 1) and 100μL was passaged into new tubes of 5mL mGAM broth. After a further 24 hours, the end-point communities were sampled and measured a final time (day 2).

For nutrient supplementation experiments (galactitol), 2.5mL mGAM buffered with 100mM MES to pH 6.2 was prepared and autoclaved in Hungate tubes. A 2x stock solution of filter-sterilised sugar in Milli-Q water (e.g. 0.2% or 2% galactitol) was prepared and de-oxygenated in the anaerobic chamber. 2.5mL of the stock solution was added into the Hungate tube to generate a 5ml total volume with the appropriate concentration of the sugar. The extended competition experiment is performed as described above, except samples were passaged again into tubes with the appropriate concentration of the sugar. Samples were left to grow for 48h instead of the standard 24h to allow utilisation of low priority sugars, such as galactitol.

For experiments with the AMR *E. coli* clinical isolate, the experiment was performed as described above, except samples were analysed using selective plating instead of flow cytometry (LB + 100μg/mL ampicillin to select for the AMR *E. coli* strain; MacConkey agar to enumerate total *E. coli* densities).

### Community preparation

All culturing was performed in a shaking incubator at 225rpm and 37ºC, and under anaerobic conditions. Constituent monocultures were grown to stationary phase, passaged into new media and grown overnight for approximately ~17 hours, and then combined into communities. The OD-600nm of the single strains was measured and the cultures were streaked anaerobically on mGAM agar to check for contamination. Communities ranged from 2 to 50 strains in size and were assembled under anaerobic conditions.

#### In vitro

For the communities containing 2-10 strains, equal volumes (100μL) of each overnight culture were combined in a Hungate tube containing 5mL mGAM to form a community. For the 49- and 50-strain communities, 20μL of each single strain was added, equating to a total volume of 1mL. The communities were made by adding 1mL of each strain anaerobically to a 50mL falcon tube, mixing, and removing 1mL to add to a fresh Hungate tube of 5mL mGAM broth. Communities were grown in the shaking incubator (37ºC, 225rpm) for 24 hours before being invaded with 10^6^ cells/mL of the pathogen.

#### In vivo

Strains were grown separately, then OD-600nm of each culture was measured. For communities containing 2-10 strains, the volume of culture containing 10^9^ cells was calculated for each individual strain, based on a standard curve of OD-600nm and flow cytometry quantification. This was added to a large falcon tube. The community mix was centrifuged (10 minutes, room temperature, 14000rpm) and the resulting pellet resuspended in 2mL anaerobic PBS and kept on ice. 200μl were gavaged into each mouse corresponding to 10^8^ cells for each strain. For the 49- or 50-strain communities, we used less cells for each strain to avoid gavaging a large total cell density into mice (as done for the *in vitro* experiments). The volume of culture containing 2*10^8^ cells was calculated for each constituent strain and they were combined. The mixture was centrifuged and the pellet resuspended in 2mL PBS. Mice were gavaged with 200μL of the inoculum. Therefore, the density of bacteria in the 200μL gavage was equivalent for the 50-strain community as for the 10-strain community (10^9^ total cells). For all inoculums, samples were taken for plating anaerobically on mGAM agar to confirm the cell density.

### Flow cytometry

Hungate tubes containing culture to be sampled were de-pressurised under anaerobic conditions using needles. Samples were removed using needles and syringes. 10μL of each sample was diluted in 90μL PBS + chloramphenicol 200μg/ml then 10μL added to a 96-well flat-bottomed plate already containing 80μL PBS + chloramphenicol 200μg/ml. The 96-well plate was left to shake at room temperature on the bench for 15 minutes to allow oxygen-dependent folding of YPet, while preventing further growth of the sample with the bacteriostatic antibiotic chloramphenicol. Next, all bacterial cells were fixed, permeabilised and fluorescently stained by adding 90μL PBS containing 4% paraformaldehyde, 0.4% Triton X-100 and 1μg/mL 4’,6-diamidino-2-phenylindole (DAPI) to each sample well and incubating the plate in the dark for 1 hour at room temperature on a microplate shaker. Prior to the measurement, 20μL AccuCheck counting beads (ThermoFisher- PBC100) was added to every sample well. Two wells containing 200μL PBS were left between sample wells, in order to prevent cross-contamination between samples. 96-well plates were run in a flow cytometer (Attune NxT Autosampler with Attune NxT Flow Cytometer, ThermoFisher) set to: acquisition volume 50μL, total draw volume 80μl, total sample volume 200μL, at a speed of 100μL/minute. Relevant spectral parameters were recorded by the flow cytometer, equipped with 405nm, 488nm and 561nm lasers. The following channels were used: DAPI staining, excitation 405nm, emission 415-465nm (‘blue’); YPet fluorescence, excitation 488nm, emission 505-515nm and 525-555nm (‘green’).

Flow cytometry data was processed using FlowJo v10.8.1 software. ‘Beads’ were gated on a linear (FSC-H; SSC-H) axis. ‘Bacteria’ were gated on a logarithmic (FSC-H; SSC-H) axis. DAPI was gated within the ‘Bacteria’ gate, on a logarithmic (Ex405-417; Ex405-495) axis. Within the DAPI gate, YPet was gated on a logarithmic (Ex488-503; Ex488-555) axis. Count statistics for beads, DAPI and YPet were exported to Microsoft Excel and pathogen density (cells/mL) was calculated.

### Mouse husbandry and experiments

Mouse experiments were performed with 6-8 weeks old germ-free wild-type C57BL/6J female mice (RRID IMSR_JAX:000664) that were bred and maintained in germ-free isolators at the Kennedy Institute, University of Oxford for more than five years. During experiments, mice were housed as pairs or trios in sterile, individually ventilated Sentry SPP cages (Allentown) with enrichment, irradiated food and autoclaved water. Cages of mice were disinfected in TecCare Ultra Hydrogen peroxide, peracetic acid solution before mice were handled using individually sterilised gloves (single use for each cage) in a biosafety cabinet that was disinfected with the same acid solution in between each treatment group. Cages of mice were randomly assigned to a group, and cages were opened in the same order each time. Researchers were not blinded because of the unbiased quantification methods of colony counting and metagenomic sequencing. Samples sizes were determined using standard practices of 7-8 mice per group distributed over 2-3 cages, and repeated the experiment three times to allow experimental independence and sufficient power for nonparametric testing. Mouse experiments were performed in accordance with the UK Animal Scientific Procedures Act (1986) under a UK Home Office licence (PPL 9127884) assessed by the Medical Sciences Division Animal Welfare and Ethical Review Body (AWERB) at the University of Oxford. Due to strictly defined humane endpoints, one mouse needed to be terminated early. In this case, we excluded data from that mouse.

Germ-free mice were gavaged with a 200μL inoculum of symbionts (see “community preparation”) at day -14 of the experiment and again on day -12 of the experiment (see scheme Fig. 3a). On day 0, overnight cultures of the pathogens (*K. pneumoniae* and *S*. Typhimurium) grown aerobically in LB containing the appropriate antibiotics were washed 3x with PBS and diluted. 10^6^ CFU of the pathogen was given to each mouse as a 100μl gavage. Feces were sampled daily after gavage of the pathogen. Fecal pellets were weighed and homogenised with a 5mm stainless steel bead in 1ml PBS (samples were shaken vigorously in 2ml Eppendorf tubes). CFUs were enumerated using selective plating (MacConkey or LB agar for *E. coli*; MacConkey + 50 μg/mL streptomycin for *S*. Typhimurium; LB + 50μg/mL carbenicillin for *K. pneumoniae*). *E. coli, K. pneumoniae* and *S*. Typhimurium, can be differentiated by color on MacConkey agar (*S*. Typhimurium cannot utilise lactose) or morphology (*K. pneumoniae* produces a capsule). Mice were euthanised 4 days after infection with the pathogen.

*S*. Typhimurium triggers inflammation to bloom in the gut (19). To allow us to focus on early colonization events prior to triggering inflammation (i.e., colonization resistance), we used an avirulent strain (SL1344 Δ*invG* Δ*ssaV*) to avoid triggering inflammation, disease and mortality (65).

### DNA isolation from feces and metagenomic sequencing

Aliquots of the feces were frozen immediately after collection (prior to homogenisation in PBS) and stored at -80°C until use. Fecal samples were thawed and resuspended in nuclease-free water, before being transferred to a lysing matrix B tube (MP Biomedicals). 3 rounds of bead beating were performed at 6 m/s for 40s. Samples were centrifuged at high speed and DNA in the supernatant was precipitated by adding sodium acetate (1/10 volume) and ice-cold ethanol (96-100%; equal volume), and left at -20°C overnight. Samples were centrifuged at high speed and the pellet was washed twice with 70% ethanol, before being dried and resuspended in nuclease-free water.

Samples were further purified using an AMPure clean-up protocol. The samples were mixed with AMpure XP beads (Beckman Coulter) and incubated at room temperature for 5 minutes, before being washed twice with 70% ethanol, using a magnet to avoid removing the DNA bound to the AMPure beads. After air drying, nuclease-free water was added to the beads, to allow collection of the purified DNA in the supernatant.

CosmosID performed metagenomic sequencing on isolated DNA. DNA libraries were prepared using the Nextera XT DNA Library Preparation Kit (Illumina) and IDT Unique Dual Indexes. Prepared libraries were sequenced on the Illumina NovaSeq 6000 platform 2x150bp (3M reads per sample). CosmosID performed bioinformatic analysis according to proprietary methods on the raw data to generate fine-grained taxonomic and relative abundance estimates (Fig. S5).

### Spent media assay

Strains were anaerobically inoculated, grown, passaged and assembled into communities as described above. Communities were grown anaerobically in the shaking incubator (225rpm, 37ºC) for 96 hours to maximise depletion of nutrients utilisable by the community. Supernatants were prepared by centrifuging the 96-hour cultures (10 minutes, 4400rpm, room temperature) and filter-sterilising the supernatant (0.2μm filter). All preparation of supernatants were done under anaerobic conditions. Two of each of the communities were assembled so that, for each community, one Hungate tube containing 5mL supernatant and another Hungate tube containing 2.5mL supernatant and 2.5mL mGAM (the re-supplemented treatment) were prepared. The supernatants were invaded with 10^6^ cells/mL of fluorescent pathogen, sampled and incubated for 24 hours as before. Samples were taken on days 0 and 1.

### Nutrient utilisation overlap using biolog assays

Utilisation of carbon sources by symbionts and the pathogens were assessed using AN Biolog Microplates™ (Biolog) according to the protocol of the manufacturer. Briefly, strains were grown in mGAM and passaged (as detailed above). The cultures were centrifuged anaerobically and washed twice in anaerobic PBS. The samples were concentrated and an aliquot was taken to measure the OD-600nm. The cells were diluted or concentrated such that a 200μl aliquot of the cells would correspond to 65% transmittance (OD 0.187; the density outlined by the manufacturer) in the 14ml volume of AN inoculating fluid provided. The concentrated cells were added to the inoculating fluid aerobically (a small amount of O_2_ is needed to oxidise the buffer) and 100μl was aliquoted into each well of the microplate. After 10min in aerobic conditions, the plates were put in an air-tight container with a GasPak EZ Container System Sachet to generate hydrogen-free anaerobic conditions. Samples were incubated at 35°C for 24h before measurement at 590nm. Since *S*. Typhimurium SL1344 is a histidine auxotroph, a prototrophic strain was made using P22 transduction (66) of the allele from a prototrophic strain of *S*. Typhimurium (ATCC 14028S). Positive clones were selected for on M9 minimal media with 50μg/ml streptomycin and re-streaked 3 times on LB plates to ensure phages are removed. Growth in the absence of exogenous histidine was validated by streaking on M9 media.

For each plate, the absorbance reading at 590nm was subtracted from the blank (no carbon source control in well A1). Each strain generates a different background signal so it is important to do this on each plate individually. Each strain was measured as three independent biological replicates and the median absorbance value was taken for each carbon source. We used a thresholding approach to determine if a strain can metabolise a given carbon source (defined as Abs 590nm >0.1 median, after blank subtraction; Fig. S10).

To predict overlap with the pathogens, whether a given strain can use a carbon source was compared to the pathogen. We assessed which percentage of carbon sources that the pathogen can use can also be used by the symbiont. To calculate overlap between pathogens and communities, we used an additive calculation approach, where if a strain is contained in a community that can use a carbon source, the entire community can use the carbon source. For simplicity, we did not treat cases where multiple species use the same nutrients within the community differently than if a given nutrient is only covered by one species.

### Genomic analysis using protein overlap

Genomic information from 50 symbiont strains and two pathogens (*S*. Typhimurium, *K. pneumoniae*) were retrieved from the PATRIC database (67) (Table S1). On this set, we applied a cluster-based analysis that groups proteins encoded in genomes into PATRIC global protein families (68). A majority of symbiont strain proteins obtained a protein family designation (clustering rate of 97.83%; 172 848 of 176 675) with protein families being populated by proteins from on average 2.44 genomes (Fig. S7).

Based on this set, we drew protein family designations for focal symbiont communities and calculated pathogen/community overlap using the same approach as for the Biolog plates above. That is, for each protein family encoded in the pathogen’s genome, we checked if it overlapped with any protein families encoded in the community.

### Whole-genome sequencing

An overnight culture of *E. coli* 19Y000018 (the AMR clinical urine isolate) was prepared in LB containing 100μg/ml ampicillin. A 1mL pellet was taken and DNA was extracted using the same ethanol precipitation and AMPure clean-up protocol detailed above in the section “DNA isolation from feces”. Source BioScience performed whole-genome Illumina sequencing on a NovaSeq 6000 to generate 10M 150bp paired end reads. Genome assembly was performed with Unicycler v0.4.8 using default settings and subjected to protein family annotation as described in “Genomic analysis using protein overlap”.

### Predictions based on biologs and protein families

#### Biolog-based predictions

The carbon source utilisation profile of the AMR *E. coli* clinical isolate was assessed using AN Biolog Microplates™ as detailed above. Next, all possible communities made of 1, 2, 3, and 5 species were identified from the 16 symbiont strains that were previously analysed for carbon source utilisation. For each community, the carbon source utilisation overlap was calculated (as detailed above; Fig. 5a). At each diversity level, communities were sorted by overlap to the AMR *E. coli* clinical isolate. Given that the symbiont *E. coli* is needed for colonization resistance in our experiments, we only included communities where the symbiont *E. coli* is present. At each diversity level, we chose the highest ranked community (defined “predicted best”) and lowest ranked community (defined “predicted worst”). If there were ties in rank, one community was randomly chosen. These communities were experimentally assessed for colonization resistance using the extended competition *in vitro* assay as detailed above.

#### Protein family-based predictions

We used the whole-genome sequence of the AMR *E. coli* isolate to calculate protein family overlap to communities of symbionts drawn from our 50-species community. As for the biolog-based predictions, we restricted the analysis to communities that contained the symbiont *E. coli* strain. Specifically, we generated 100,000 different communities randomly, each containing the symbiont *E. coli* and a total of 2, 3, 5, or 10 species from the 50 species pool. We ranked the communities according to their pathogen overlap at each diversity level and selected the highest and lowest ranked communities for experimental validation. If ties occurred, we chose the communities at random, as for the biolog-based predictions.

### Statistical analysis

All graphs were made and statistical analysis carried out in Prism v9.4.1 (GraphPad). Protein family overlap analyses and protein family-based predictions were computed in R version v4.0.5 (69). Figure legends indicate the statistical test used and the sample sizes. Nonparametric tests were used to avoid the assumptions of normality.

### Ethical statement

Mouse experiments were performed in accordance with the UK Animal Scientific Procedures Act (1986) under a UK Home Office licence (PPL 9127884) assessed by the Medical Sciences Division Animal Welfare and Ethical Review Body (AWERB) at the University of Oxford. Mice were euthanised using cervical dislocation followed by exsanguination as a confirmation of death.

## Supplementary Material

Supplementary Material

## Data Availability

All data used to generate the plots is available via DRYAD (70). All code is available via GitHub (71). Metagenomic sequencing and whole-genome sequencing of *E. coli* 0018 (NCBI accession number JAVXZX000000000) has been deposited in SRA under the BioProject PRJNA1021490. There is no restriction on use of the data, materials, or code, with the exception of *E. coli* strain 19Y000018 that is protected by an MTA which requires permission from the Nottingham University Hospitals Pathogen Bank (https://www.nuh.nhs.uk/pathogen-industry/). A Materials Design Analysis Reporting checklist is supplied with the publication.

## One sentence summary

Diverse communities of gut bacteria collectively limit pathogen colonization by blocking nutrient access.
