# Supplementary material for: Microbiome diversity protects against pathogens by nutrient blocking
Source: Science. Author manuscript; Available in PMC 2024 Oct 8. (PMC7616675; doi:10.1126/science.adj3502)
Supplement: Supplementary Material [file EMS199119-supplement-Supplementary_Material.zip › science.adj3502_sm.pdf]

Science
AAAS

Supplementary Materials for

Microbiome diversity protects against pathogens by nutrient blocking

Frances Spragge *et al.*

Corresponding authors: Olivier Cunrath, olivier.cunrath@unistra.fr; Kevin R. Foster, kevin.foster@biology.ox.ac.uk

Science **382**, eadj3502 (2023)
DOI: 10.1126/science.adj3502

The PDF file includes:

Figs. S1 to S13
Tables S1 to S7
References

Other Supplementary Material for this manuscript includes the following:

MDAR Reproducibility Checklist

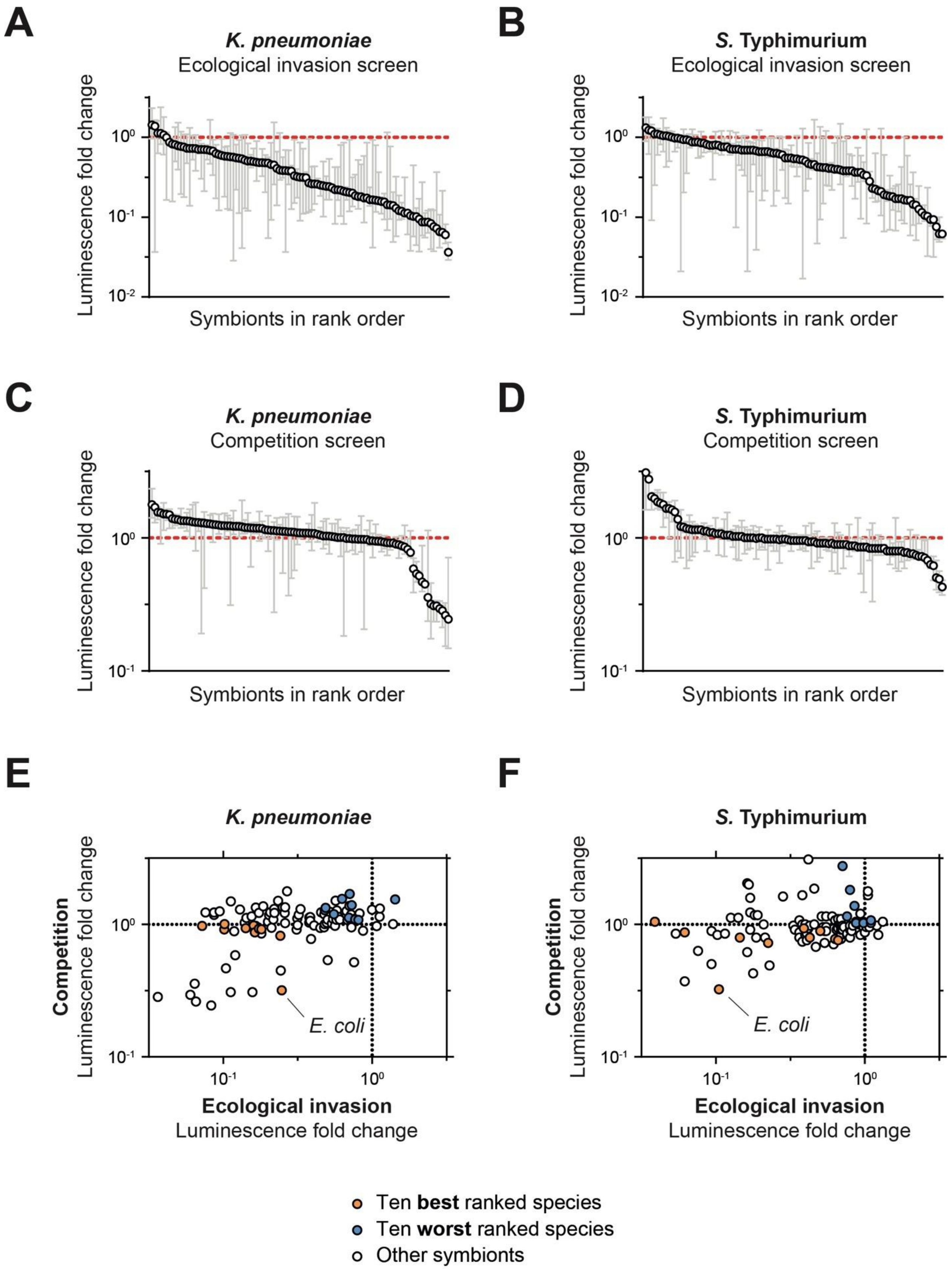
A
K. pneumoniae
Ecological invasion screen
Luminescence fold change
Symbionts in rank order
B
S. Typhimurium
Ecological invasion screen
Luminescence fold change
Symbionts in rank order
C
K. pneumoniae
Competition screen
Luminescence fold change
Symbionts in rank order
D
S. Typhimurium
Competition screen
Luminescence fold change
Symbionts in rank order
E
K. pneumoniae
Competition
Luminescence fold change
E. coli
Ecological invasion
Luminescence fold change
F
S. Typhimurium
Competition
Luminescence fold change
E. coli
Ecological invasion
Luminescence fold change
Ten best ranked species
Ten worst ranked species
Other symbionts

Fig. S1.

Human gut symbiont strains vary in their ability to inhibit growth of *K. pneumoniae* and *S.* Typhimurium in the luminescence screen. **A-D)** Waterfall plots show the luminescence median fold change (log ratio of the control luminescence divided by the treatment luminescence) of the pathogen in combination with each human gut symbiont tested in the luminescence screen. Results for *K. pneumoniae* are shown in **(A)** and **(C)** and for *S.* Typhimurium shown in **(B)** and **(D).** Results of the ecological invasion assay are shown in **(A)** and **(B)** and the competition assay in **(C)** and **(D).** Black circles represent the median value for each symbiont (*N*=3-10 biological replicates from independent experiments). Grey vertical lines represent range bars. **E-F)** Correlation of the results of the ecological invasion and competition assays of the luminescence screen. Each circle represents the median value for a symbiont (*N*=3-10 biological replicates from independent experiments). Results for *K. pneumoniae* shown in **(E)** and *S.* Typhimurium in **(F).** The sum of the ranks of the competition and the ecological invasion assays were used to create an overall ranking of symbionts for each pathogen. The best 10 ranked species are shown in orange and the worst 10 shown in blue, with the added criteria that they have a category 1 safety level (see Methods). Strains with the most negative competition and invasion values inhibited the growth of the pathogen most strongly compared to the media-only control.

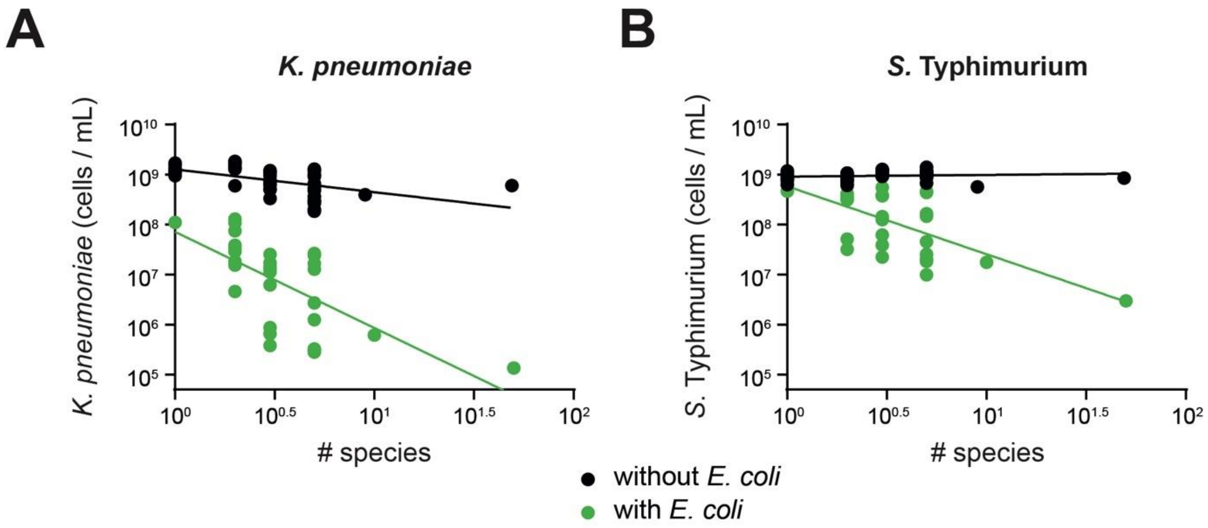

Fig. S2.

Community diversity negatively correlates with pathogen abundance. A-B) As the number of symbiont strains increases, pathogen density on day 2 of the extended competition decreases (x axis on a log scale). Data for *K. pneumoniae* shown in **(A)** and *S.* Typhimurium in **(B).** Each circle represents the median value of a community tested in the extended competition assay (data from **Fig. 2C-D**). Communities in green contain *E. coli*, communities in black do not contain *E. coli*. Linear regression of log-transformed data: **(A)** R^2=0.4296, non-zero slope for *E. coli* communities (F test, p<0.0001). R^2=0.3103, non-zero slope for communities without *E. coli* (F test, p<0.0001). Moreover, slopes of the two regressions are significantly different from each other (F test, p=0.0001). **(B)** R^2=0.4234, non-zero slope for *E. coli* communities (F test, p<0.0001). R^2=0.01378, slope not different to zero for communities without *E. coli* (F test, p=0.4218). Slopes of the two regressions are again significantly different from each other (F test, p<0.0001).

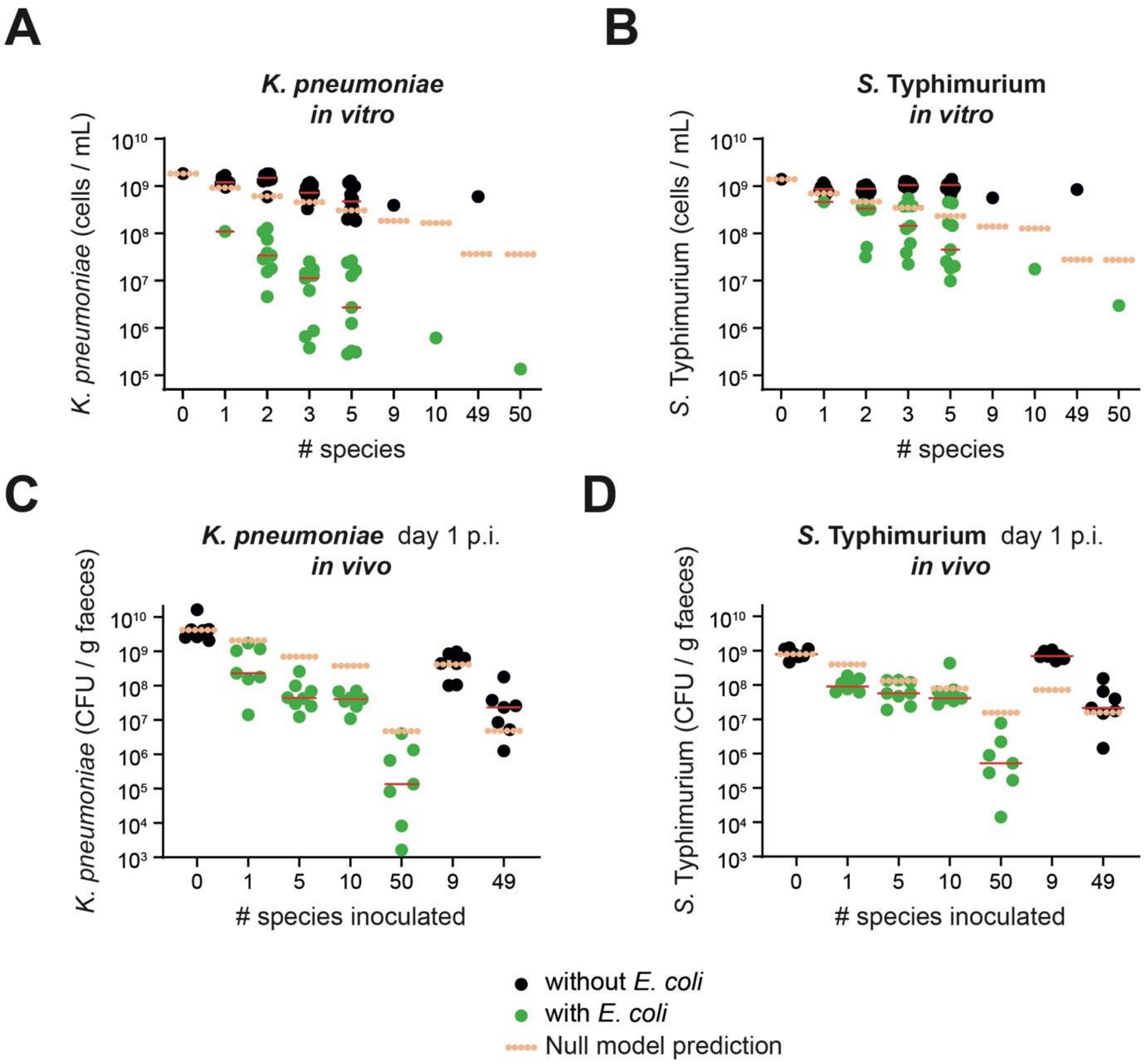

Fig. S3.

Comparison to a null model where the effect of each species proportionally restricts pathogen growth in an additive manner. We compared our experimental data to a null model where the effect of colonization resistance simply scales according to the number of species in the community. Specifically, we took the abundance of the pathogen at the end of the experiment when the pathogen is alone (ie, n=0) and multiplied it by 1/(n) where n refers to the number of species that contribute to the overall carrying capacity of the system (including the pathogen). This value is plotted in as beige dotted lines for both *in vitro* data from **Fig. 2C-D** (panels A and B; dots indicate median values of each community) and *in vivo* data from **Fig. 3D-E** (panels C and D; dots indicate individual mice). **A,C**) Data for *K. pneumoniae*, **B,D**) Data for *S.* Typhimurium. Communities containing *E. coli* are shown in green and communities without *E. coli* are shown in black. Red lines indicate medians. In all cases, the deviation between the null model and data increases at higher diversities, so long *E. coli* is present in the high diversity communities. This effect is also statistically significant in all cases: we compare the ratio of predicted to observed pathogen abundance for 1 species data to that from the 50 species case (Two-tailed Mann-Whitney U tests; p=0.0070 for **panel A**; p=0.0070 for **panel B**; p=0.0041 for **panel C**; p=0.0262 for **panel D**).

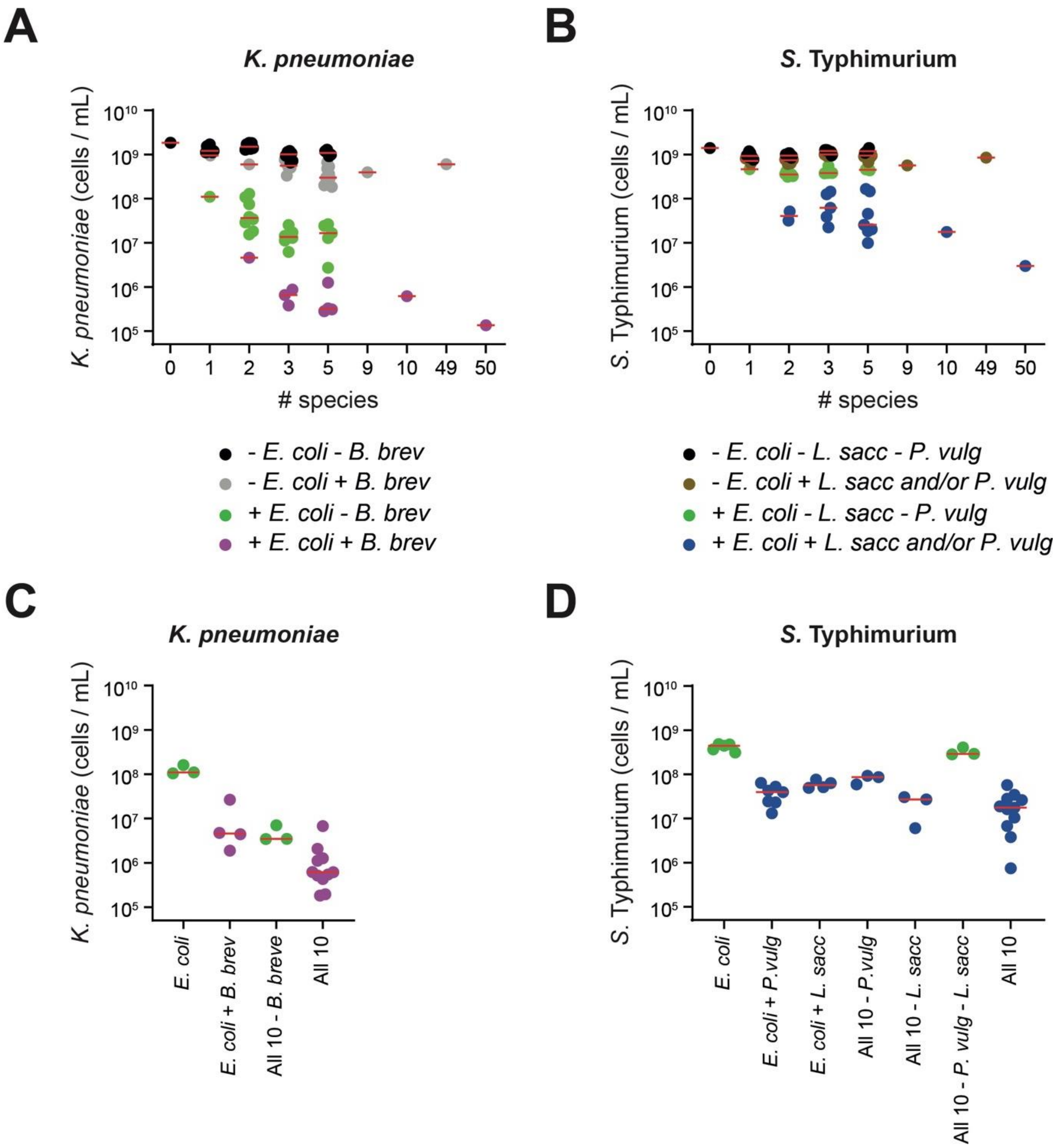

Fig. S4.

Combinations of multiple species are important for colonization resistance to each pathogen. A-B) Equivalent figures to **Fig. 2C-D**, except the presence or absence of *B. breve* within communities are shown in **(A)** (grey for communities with *B. breve* but without *E. coli* and purple for communities with *B. breve* and *E. coli*), and the presence or absence of *L. saccharolyticum* and/or *P. vulgatus* shown in **(B)** (brown for communities with *L. saccharolyticum* and/or *P. vulgatus* but not *E. coli*, and blue for communities with *L. saccharolyticum* and/or *P. vulgatus* and *E. coli*). Results for *K. pneumoniae* are shown in **(A)** and for *S.* Typhimurium shown in **(B).** In **(A-B)**, “+” and “-” refer to the presence or absence of species rather than the addition or subtraction of a species. Horizontal red lines depict the median of the communities at a particular diversity level containing the species indicated in the legend. Each circle represents the median pathogen abundance measured for a community on day 2 of the extended competition assay. **C-D)** Drop-out experiments verify the context-

dependent effect of key members on colonization resistance. *N*=3-11 biological replicates from independent experiments. In **(C-D)**, "+" and "-" refer to the addition or subtraction of a species. Results for *K. pneumoniae* are shown in **(C)** and for *S.* Typhimurium shown in **(D).** Horizontal red lines depict the median of the replicates for a particular community. See **Table S1** for species name abbreviations.

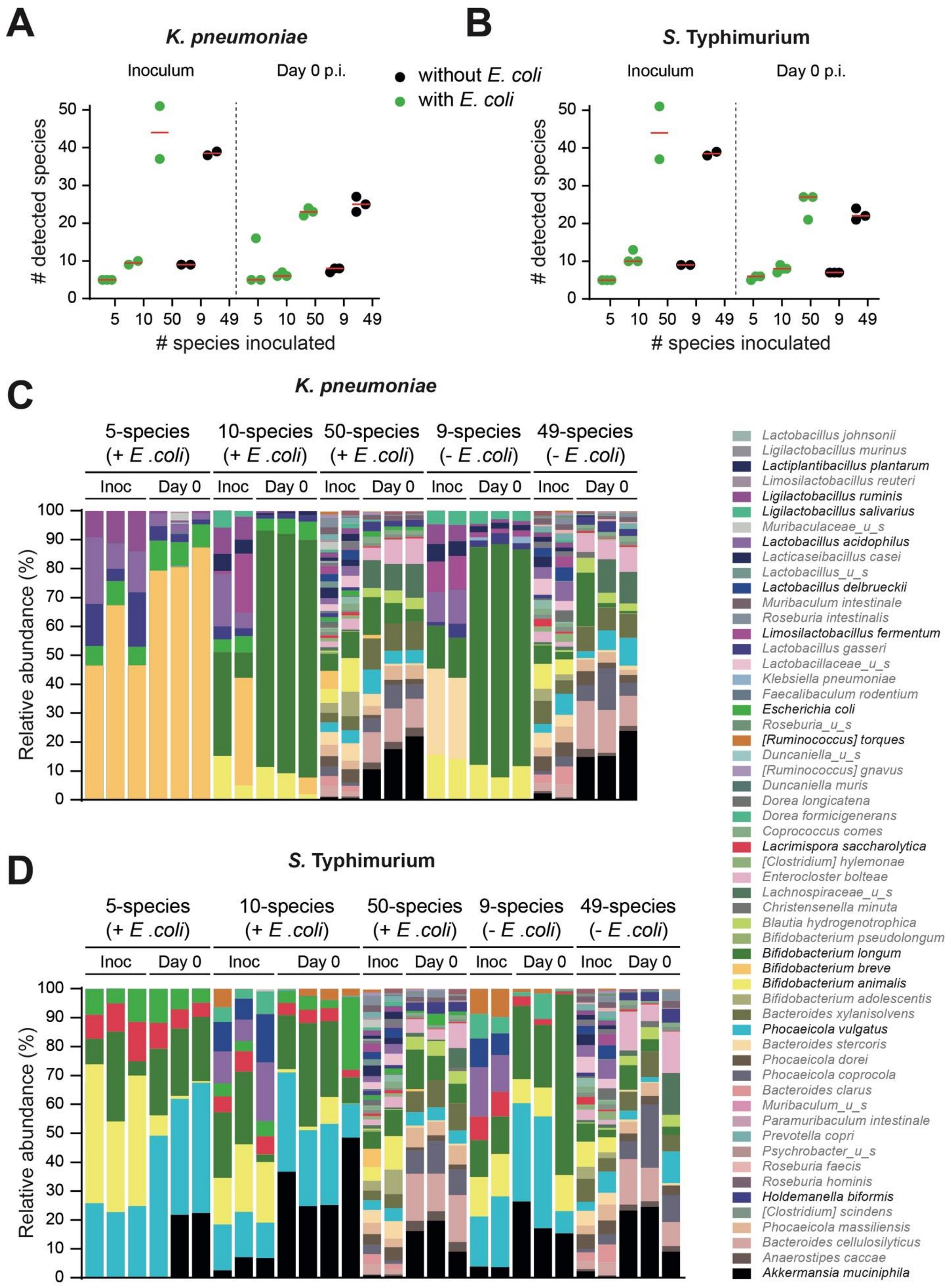
A
K. pneumoniae
Inoculum
Day 0 p.i.
without E. coli
with E. coli
detected species
species inoculated
5 10 50 9 49 5 10 50 9 49
B
S. Typhimurium
C
K. pneumoniae
5-species (+ E .coli)
10-species (+ E .coli)
50-species (+ E .coli)
9-species (- E .coli)
49-species (- E .coli)
Inoc
Day 0
Relative abundance (%)
D
S. Typhimurium
Lactobacillus johnsonii
Ligilactobacillus murinus
Lactiplantibacillus plantarum
Limosilactobacillus reuteri
Ligilactobacillus ruminis
Ligilactobacillus salivarius
Muribaculaceae_u_s
Lactobacillus acidophilus
Lacticaseibacillus casei
Lactobacillus_u_s
Lactobacillus delbrueckii
Muribaculum intestinale
Roseburia intestinalis
Limosilactobacillus fermentum
Lactobacillus gasseri
Lactobacillaceae_u_s
Klebsiella pneumoniae
Faecalibaculum rodentium
Escherichia coli
Roseburia_u_s
[Ruminococcus] torques
Duncaniella_u_s
[Ruminococcus] gnavus
Duncaniella muris
Dorea longicatena
Dorea formicigenerans
Coprococcus comes
Lacrimispora saccharolytica
[Clostridium] hylemonae
Enterocloster bolteae
Lachnospiraceae_u_s
Christensenella minuta
Blautia hydrogenotrophica
Bifidobacterium pseudolongum
Bifidobacterium longum
Bifidobacterium breve
Bifidobacterium animalis
Bifidobacterium adolescentis
Bacteroides xylanisolvens
Phocaeicola vulgatus
Bacteroides stercoris
Phocaeicola dorei
Phocaeicola coprocola
Bacteroides clarus
Muribaculum_u_s
Paramuribaculum intestinale
Prevotella copri
Psychrobacter_u_s
Roseburia faecis
Roseburia hominis
Holdemanella biformis
[Clostridium] scindens
Phocaeicola massiliensis
Bacteroides cellulosilyticus
Anaerostipes caccae
Akkermansia muciniphila

Fig. S5.

Metagenomic sequencing shows that germ-free mice gavaged with more diverse communities were colonized with a higher number of bacterial strains. A-B) Number of detected bacterial species **(**above a relative abundance threshold of 0.1%) in the inoculum given to the mice and in mouse feces 14 days after the first inoculum gavage (Day 0 post infection; p.i.). Mice were given 2 identical gavages containing symbiont communities 2 days apart; the first inoculum was sequenced as a representative. Each inoculum data point depicts an independent experiment and each day 0 data point indicates a representative mouse from each cage. Horizontal red lines represent median values of the replicates at each diversity level. (*N*=2-3 for the inoculum, *N*=3-4 for fecal samples; data indicates biological replicates of a representative mouse from each cage, derived from at least two independent experiments). Green symbols represent communities that contain *E. coli*, black symbols represent communities without *E. coli*. **C-D)** Relative abundance plot of symbiont strains in the inoculum and mouse feces using metagenomic sequencing data. Data for mice challenged with *K. pneumoniae* shown in **(C)** and *S.* Typhimurium in **(D).** The 10 best ranked strains from the luminescence screen for each pathogen are shown in black writing (other detected species in grey writing).

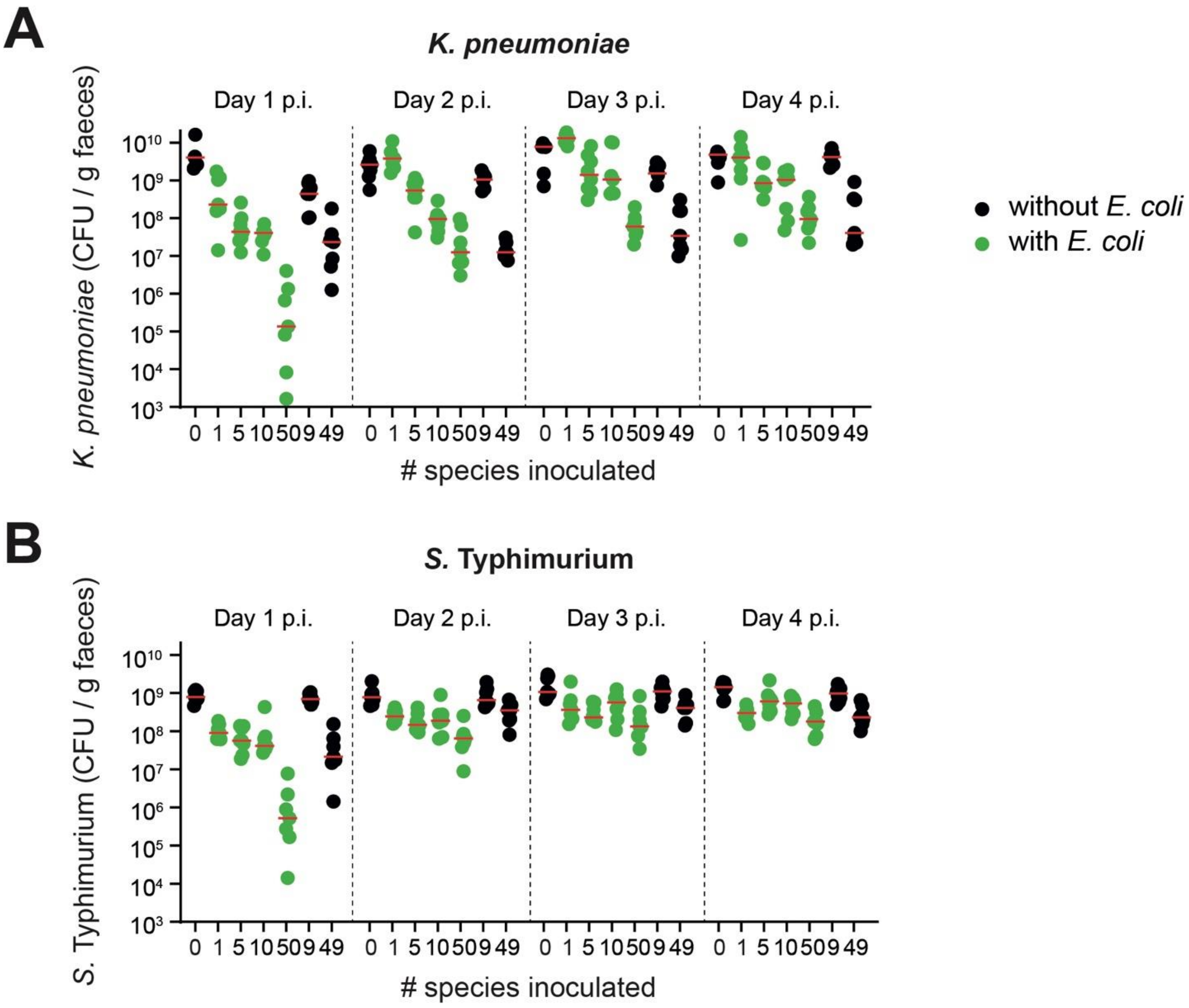

Fig. S6.

Pathogen abundance *in vivo* at later timepoints. A-B) Mice were gavaged with *K. pneumoniae* **(A)** or *S.* Typhimurium **(B)** on Day 0. Each symbol represents a fecal sample from 1 mouse. Pathogen abundances were determined by selective plating aerobically on LB agar + carbenicillin (*K. pneumoniae*) or LB agar + streptomycin (*S.* Typhimurium). Horizontal red lines represent median values of the replicate mice tested at each diversity level. Communities containing *E. coli* are shown in green whereas communities without *E. coli* are in black. *N*=7-8 biological replicates of mice per group in cages of 2-3 mice; 2-3 independent experiments. The day 1 post infection (p.i.) data is the same as in **Fig. 3D-E**.

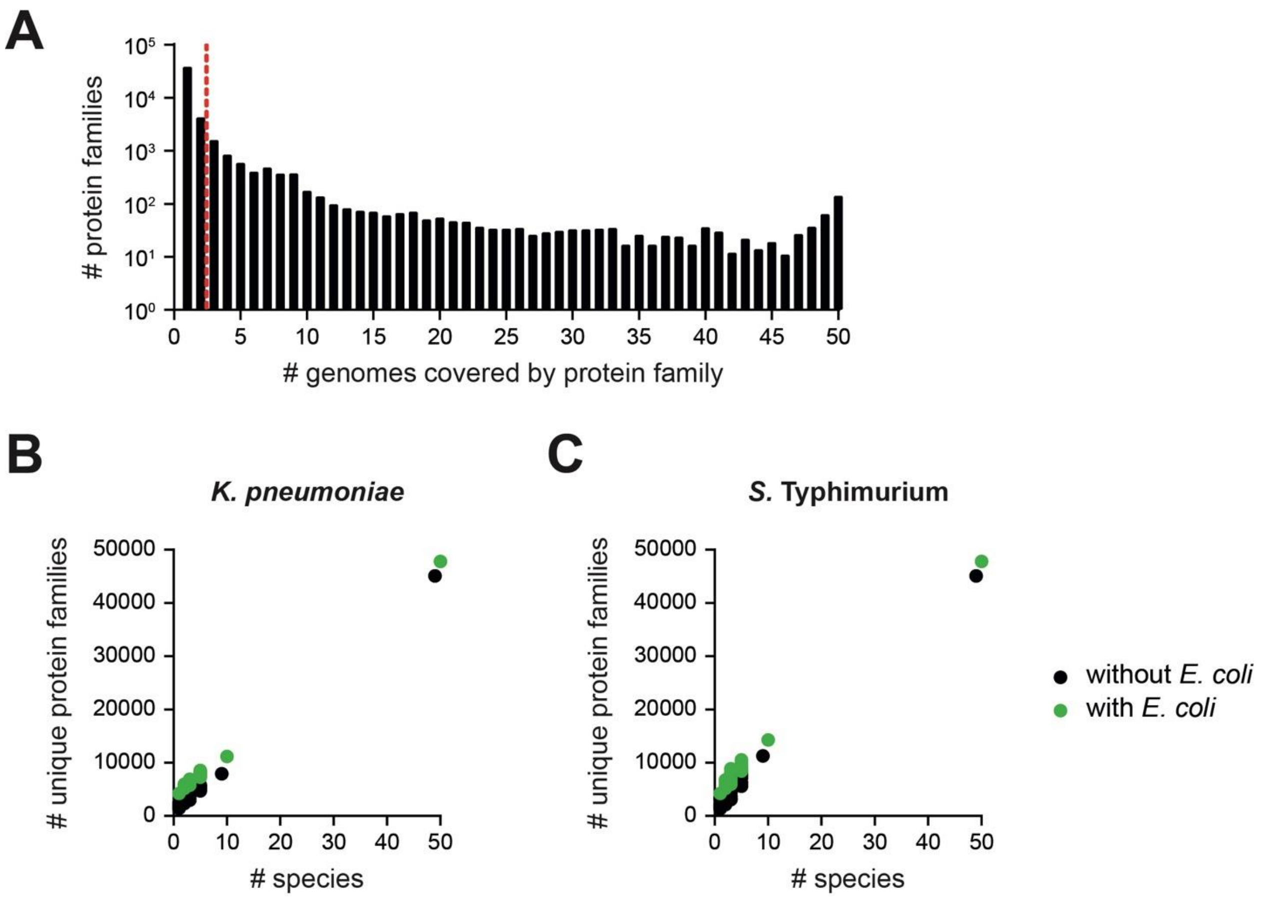

Fig. S7.

The number of protein families increase proportional to community diversity. A) Histogram showing the distribution of protein families among the 50 species subset used for protein family analysis. The vertical red dashed line represents the average number of genomes out of the 50 strains that share a particular protein family (2.44 genomes). There is an average of 3.22 proteins in each protein family. The histogram shows that many protein families are unique to a strain while others (141) are shared between all 50 strains. **B-C)** The number of protein families covered by a community increases as the number of strains in the community increases. Results for *K. pneumoniae* shown in **(B)** and for *S.* Typhimurium in **(C).** Community IDs taken from **Fig. 2C-D**. Each circle represents a different community. Green circles depict communities containing *E. coli*, while black circles are communities without *E. coli*.

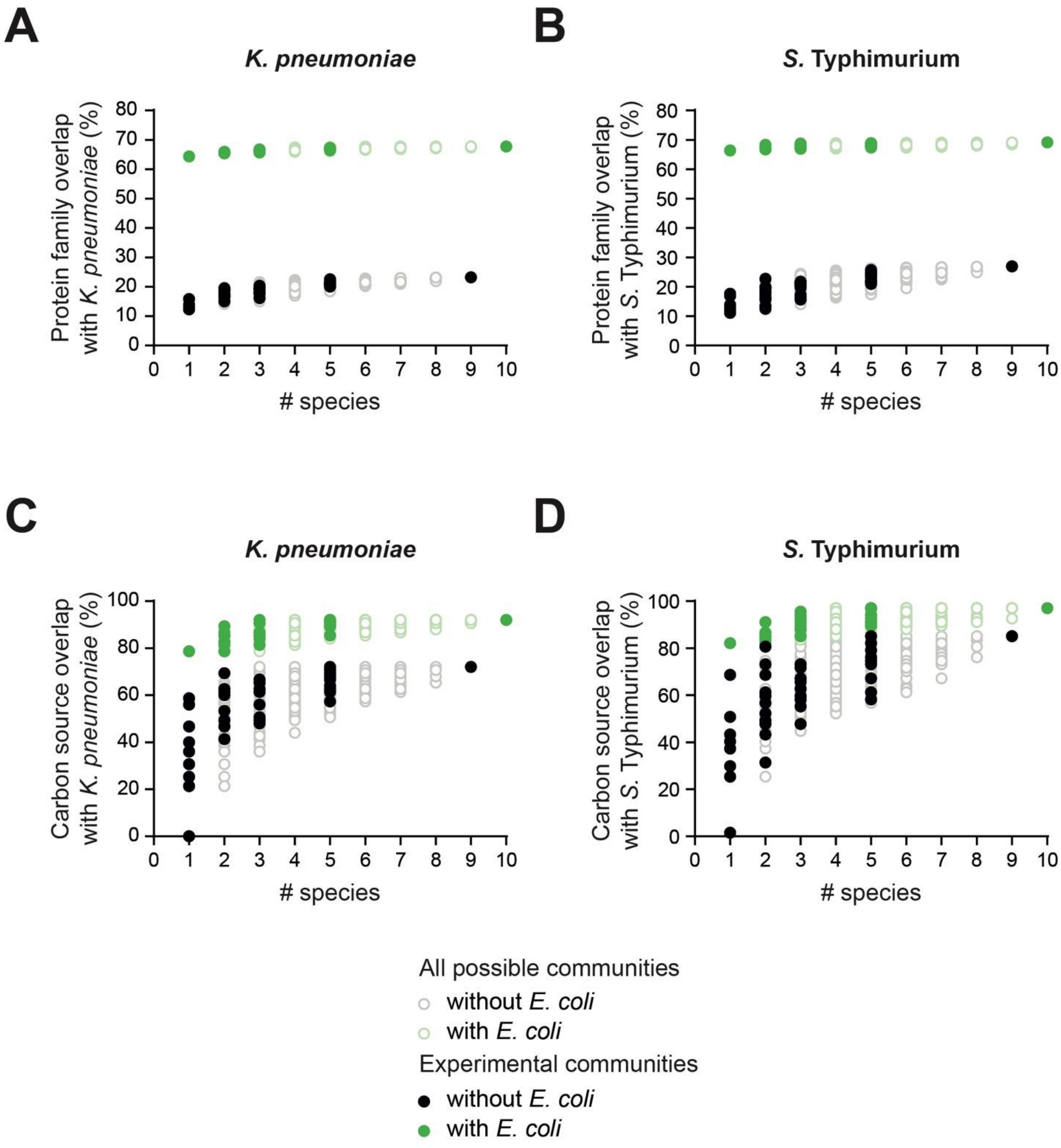

Fig. S8.

The randomly chosen communities used in the *in vitro* experiments are representative of all possible combinations of the 10 best-ranked species. A-B) Experimental communities contain representative protein family overlap to the pathogens compared to all possible combinations of the 10 best-ranked species. All possible combinations of communities are depicted by unfilled circles, experimentally tested communities are shown as filled circles. Communities in green contain *E. coli* while communities in black do not contain *E. coli*. Data for *K. pneumoniae* shown in **(A)** and for *S.* Typhimurium in **(B);** data from **Fig. 2C-D. C-D)** Experimental communities contain representative carbon source utilization overlap to the pathogens compared to all possible combinations of the 10 best-ranked species. All possible combinations of communities are depicted by unfilled circles, experimentally tested

communities are shown as filled circles. Communities in green contain *E. coli* while communities in black do not contain *E. coli*. Data for *K. pneumoniae* shown in **(C)** and for *S.* Typhimurium in **(D).**

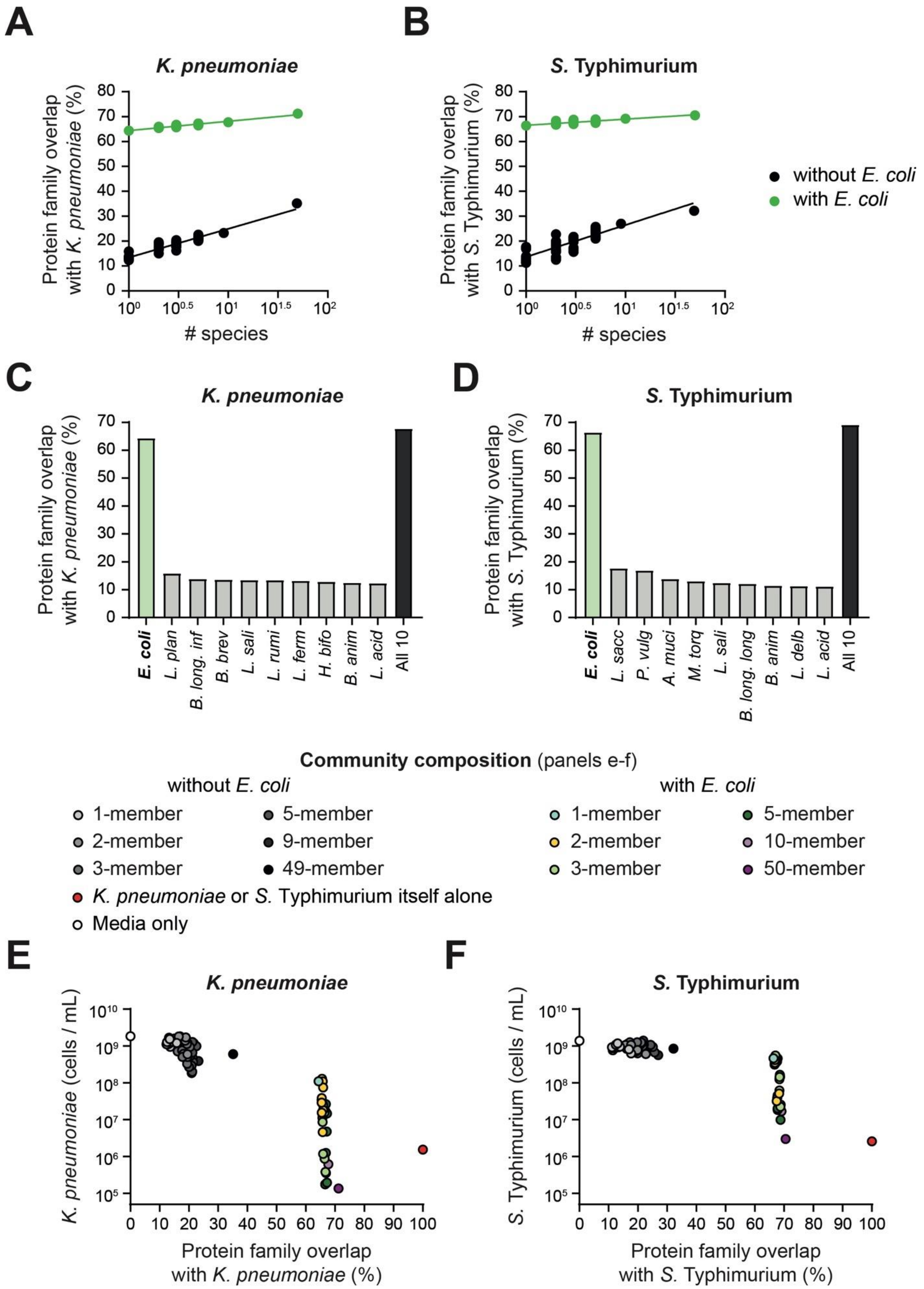
A
K. pneumoniae
Protein family overlap with K. pneumoniae (%)
species
B
S. Typhimurium
Protein family overlap with S. Typhimurium (%)
without E. coli
with E. coli
C
K. pneumoniae
E. coli
L. plan
B. long. inf
B. brev
L. sali
L. rumi
L. ferm
H. bifo
B. anim
L. acid
All 10
D
S. Typhimurium
E. coli
L. sacc
P. vulg
A. muci
M. torq
L. sali
B. long. long
B. anim
L. delb
L. acid
All 10
Community composition (panels e-f)
without E. coli
1-member
2-member
3-member
5-member
9-member
49-member
with E. coli
1-member
2-member
3-member
5-member
10-member
50-member
K. pneumoniae or S. Typhimurium itself alone
Media only
E
K. pneumoniae
K. pneumoniae (cells / mL)
Protein family overlap with K. pneumoniae (%)
F
S. Typhimurium
S. Typhimurium (cells / mL)
Protein family overlap with S. Typhimurium (%)

Fig. S9.

Protein family overlap between single strains or communities and the pathogen shows that both diversity and key members (*E. coli*) are important in explaining predicted colonization resistance. A-B) As community diversity increases, protein family overlap with the pathogen increases. Results for *K. pneumoniae* shown in **(A)** and for *S.* Typhimurium in **(B).** Each circle represents a community (communities from **Fig. 2C-D).** Green circles depict communities containing *E. coli*, while black circles are communities without *E. coli*. Linear regression of log-transformed data: **(A)** $R^2=0.9350$, non-zero slope for *E. coli* communities (F test, $p<0.0001$). $R^2=0.9182$, non-zero slope for communities without *E. coli* (F test, $p<0.0001$). **(B)** $R^2=0.6825$, non-zero slope for *E. coli* communities (F test, $p<0.0001$). $R^2=0.7484$, non-zero slope for communities without *E. coli* (F test, $p<0.0001$). **C-D)** Bar chart showing the protein family overlap with the pathogen for the individual 10 best-ranked strains. Results for *K. pneumoniae* shown in **(C)** and for *S.* Typhimurium in **(D).** The bar for *E. coli* is shown in green and the other strains in grey. The predicted protein family overlap for all 10 strains is shown in dark grey. See **Table S1** for species name abbreviations. **E-F)** As community cluster overlap with the pathogen increases, the pathogen abundance on day 2 of the extended competition decreases. Colored circles depict communities containing *E. coli*, while black circles represent communities without *E. coli* (data from **Fig. 2C-D).** Color or greyscale gradients indicate the diversity of the community. The red circles represent the isogenic wildtype pathogens. Results for *K. pneumoniae* shown in **(E)** and for *S.* Typhimurium in **(F).**

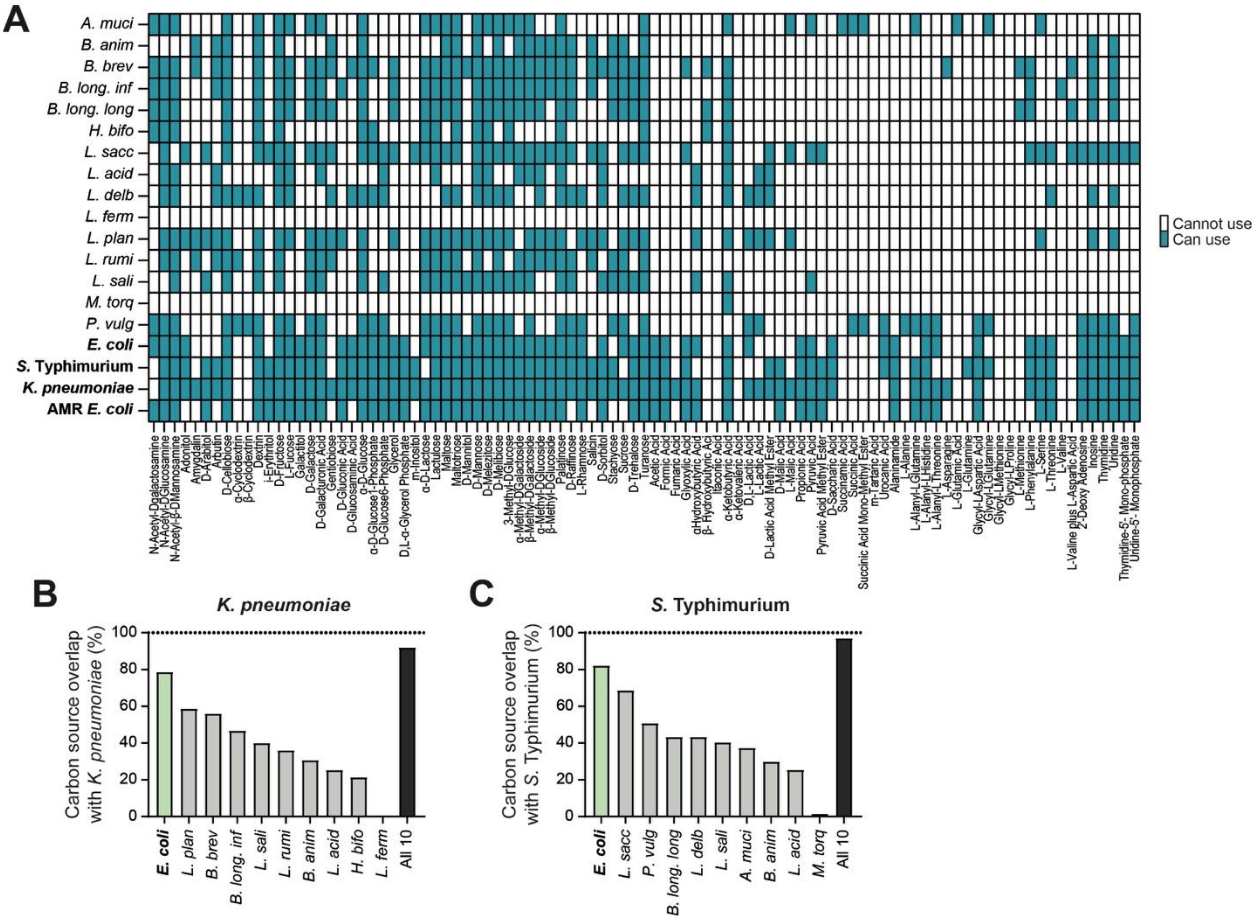

Fig. S10.

Individual carbon source utilization profiles of the 10 best-ranked symbiont strains for *K. pneumoniae* and *S.* Typhimurium (16 symbiont strains total) and their overlap with the pathogens. (A) The x-axis lists the 95 individual nutrients tested in the AN Biolog MicroPlates and the y-axis shows the 16 symbiont strains and the 3 pathogens (*K. pneumoniae*, *S.* Typhimurium, AMR *E. coli*). The pathogens are highlighted in bold on the y-axis. Nutrients shaded in blue could be used by a strain whereas those in white could not be used as defined by a threshold of background-subtracted Abs_{590nm}. Data used to apply thresholding is derived from the median value of three biological replicates of AN Biolog measurements, coming from three independent experiments. **B-C)** Bar charts showing carbon source utilization overlap (%) of the 10 best ranked individual strains with the pathogens. Results for *K. pneumoniae* in **(B)** and *S.* Typhimurium in **(C).** *E. coli* is shown in green and the other individual strains in grey. The predicted utilization of all 10 best-ranked symbionts together is shown in dark grey. The percentage overlap with the pathogen is calculated as the proportion of the number of nutrients able to be used by the pathogen that can also be used by a particular symbiont strain or community. See **Table S1** for species name abbreviations.

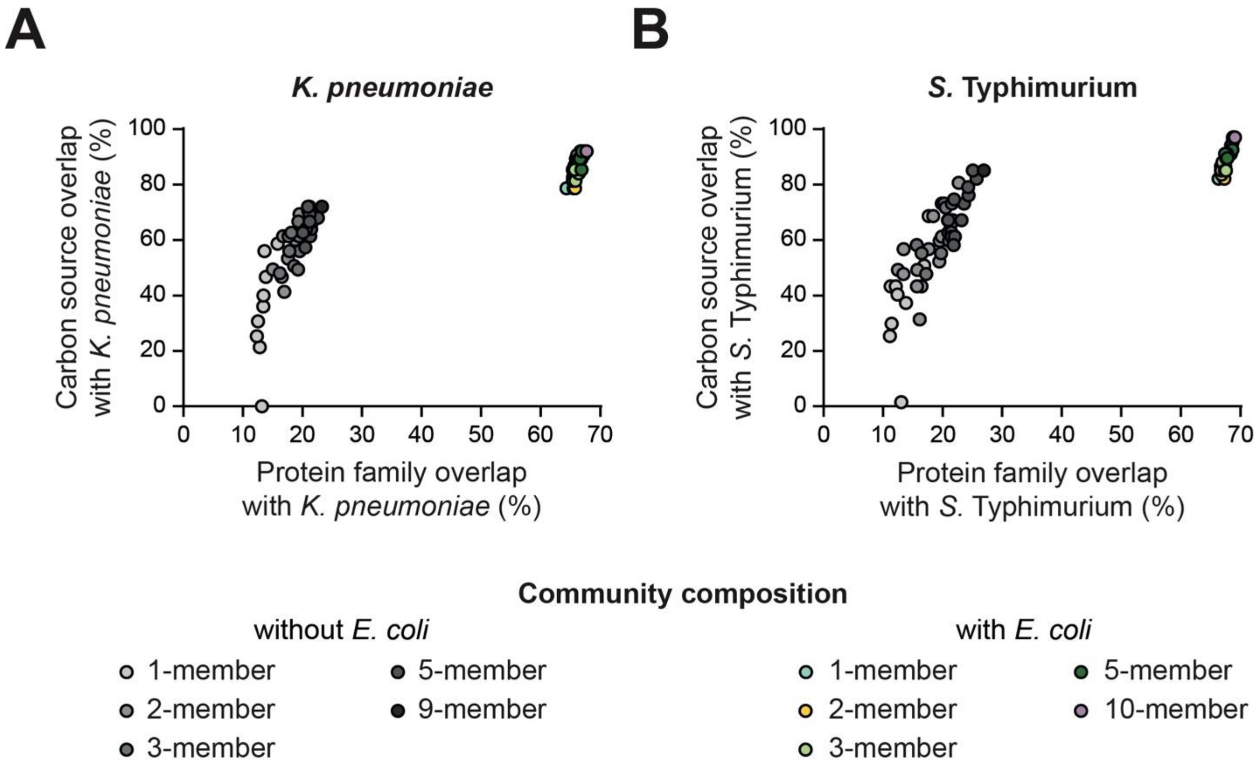

Fig. S11.

The protein family overlap and carbon source overlap prediction approaches are positively correlated. A-B) Correlation between protein cluster percentage overlap with the pathogen and carbon source utilization percentage overlap with the pathogen. Results for *K. pneumoniae* in **(A)** and *S.* Typhimurium in **(B).** Communities shown in color contain *E. coli* and those in black do not. Gradients of color or greyscale intensity show community diversity. **(A)** R^2=0.6119, slope significantly different than 0 by an F test for communities with *E. coli* ($p<0.0001$). R^2=0.6838, slope significantly different than 0 by an F test for communities without *E. coli* ($p<0.0001$). **(B)** R^2=0.8204, slope significantly different than 0 by an F test for communities with *E. coli* ($p<0.0001$). R^2=0.7027, slope significantly different than 0 by an F test for communities without *E. coli* ($p<0.0001$). The communities are the same as those in **Fig. 2C-D.** Percentage overlap calculated as the proportion of shared carbon source use or shared protein families with the pathogen. Values for communities calculated in an additive way based on the profiles of individual strains. Each data point represents a community.

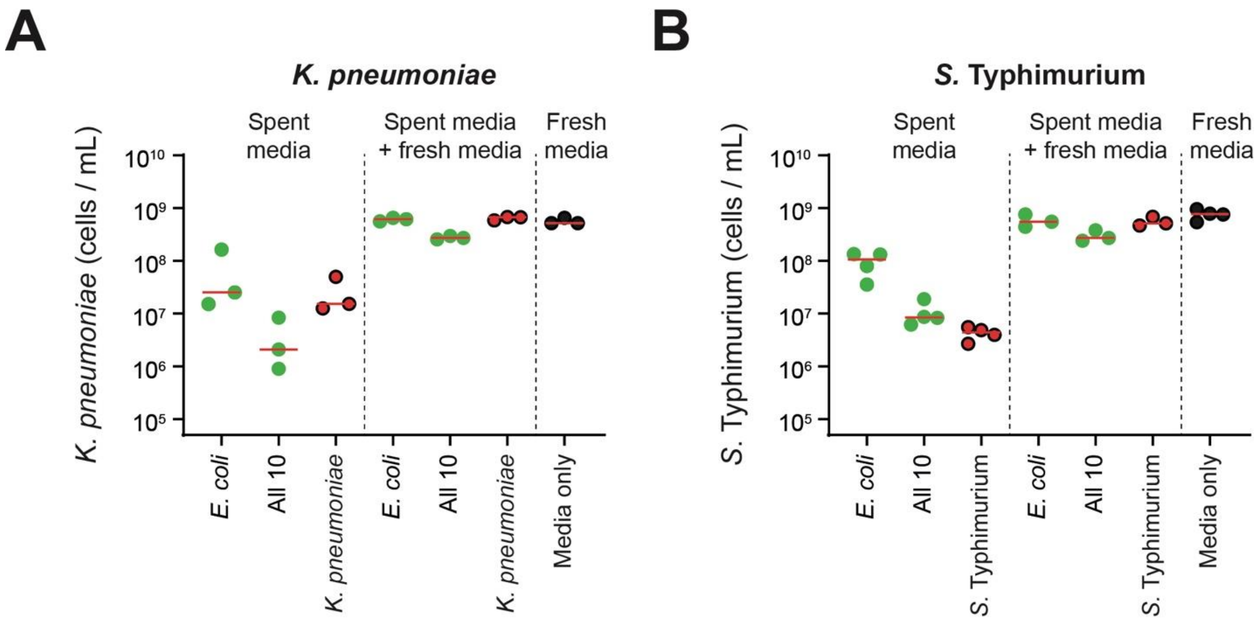

Fig. S12.

Spent media experiment. Communities were assembled and grown for 96 hours and the pathogen invaded into the spent media or re-supplemented spent media (half volume spent media and half volume nutrient media). Pathogen density measured by flow cytometry 24 hours after pathogen invasion (day 1). Results for *K. pneumoniae* shown in **(A)** and *S.* Typhimurium in **(B).** *N*=3-4 biological replicates from different independent experiments per treatment. Horizontal red lines show the median of the replicates.

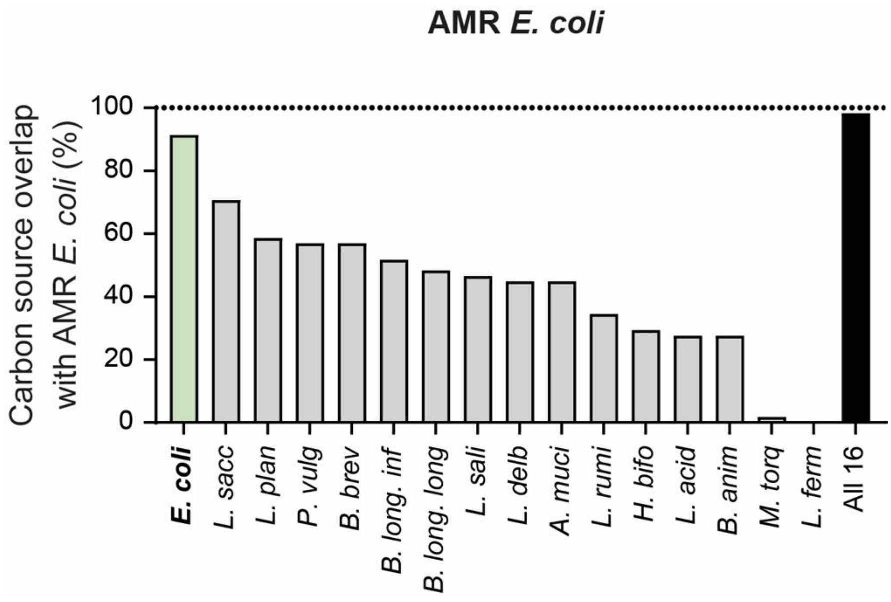

Fig. S13.

Carbon source utilization overlap with AMR *E. coli*. Bar chart showing carbon source utilization overlap (%) of the 16 best ranked individual strains (for *K. pneumoniae* and *S.* Typhimurium) with the AMR *E. coli* strain. The *E. coli* symbiont is shown in green and the other individual strains in grey. All 16 symbiont strains together are shown in black. The percentage overlap with the pathogen is calculated as the proportion of the number of nutrients that can be used by AMR *E. coli* that can also be used by a particular symbiont strain. Data used to apply thresholding is derived from the median value of 3 biological replicates of AN Biolog measurements, coming from three independent experiments.

Table S1.

Strains used in this study. *Relevant resistances only. Streptomycin= >50 ug/mL; carbenicillin=>50µg/mL; ampicillin=>100µg/mL; kanamycin=>50µg/mL; tetracycline=>50µg/mL. **According to European biosafety designation. *** Leibniz Institute DSMZ-German Collection of Microorganisms and Cell Cultures, Inhoffenstraße 7, 38124 Braunschwe, Science Campus Braunschweig-Süd, Germany. **** ATCC (American Type Culture Collection), 10801 University Boulevard, Manassas, Virginia 20110-2209, United States.

**Strain**	**Abbrev.**	**Relevant genotype ***	**Internal ID**	**PATRIC ID**	**Collection ID**	**Hazard group****	**100-strain screen**	**50-strain community**	***K. pneumoniae* 10 best-ranked**	***S.* Typhimurium 10 best-ranked**	**Source**
Actinomyces odontolyticus			NT5039	411466.7	DSM43331	2	+				Nassos Typas
Akkermansia muciniphila	*A.muci*		F3	349741.6	DSM22959	1	+	+		+	DSMZ***
Anaerostipes caccae			F2	105841.35	DSM14662	1	+	+			DSMZ
Bacteroides caccae			B1	411901.7	DSM19024	2	+				DSMZ
Bacteroides cellulosilyticus			B2	537012.5	DSM14838	1	+	+			DSMZ
Bacteroides clarus			NT5052	762984.10	DSM22519	1	+	+			Nassos Typas

**Strain**	**Abbrev.**	**Relevant genotype ***	**Internal ID**	**PATRIC ID**	**Collection ID**	**Hazard group****	**100-strain screen**	**50-strain community**	***K. pneumoniae* 10 best-ranked**	***S.* Typhimurium 10 best-ranked**	**Source**
Bacteroides eggerthii			B13	483216.6	DSM20697	2	+				DSMZ
Bacteroides fragilis enterotoxigenic 20656-2-1			NT5033	817.95	ATCC43860	2	+				Nassos Typas
Bacteroides fragilis 3_1_12			NT5057	457424.5	HM-20	2	+				Nassos Typas
Bacteroides fragilis CL07T12C05			NT5059	997883.3	HM-710	2	+				Nassos Typas
Bacteroides fragilis CL05T00C42			NT5060	997880.3	HM-711	2	+				Nassos Typas
Bacteroides fragilis CL05T12C13			NT5062	997881.3	HM-712	2	+				Nassos Typas
Bacteroides fragilis CL03T00C08			NT5063	997878.3	HM-713	2	+				Nassos Typas

**Strain**	**Abbrev.**	**Relevant genotype ***	**Internal ID**	**PATRIC ID**	**Collection ID**	**Hazard group****	**100-strain screen**	**50-strain community**	***K. pneumoniae* 10 best-ranked**	***S.* Typhimurium 10 best-ranked**	**Source**
Bacteroides fragilis CL03T12C07			NT5061	997879.7	HM-714	2	+				Nassos Typas
Bacteroides fragilis nontoxigenic			B4	272559.17	DSM2151	2	+				DSMZ
Bacteroides ovatus			B6	411476.11	DSM1896	2	+				DSMZ
Bacteroides stercoris VPI B5-21			B8	46506.156 2	DSM19555	1	+	+			DSMZ
Bacteroides stercoris CC31F			NT5055	1073351.3	HM-1036	2	+				Nassos Typas
Bacteroides thetaiotaomicron			B9	226186.12	DSM2079	2	+				DSMZ
Bacteroides uniformis			B10	820.37	DSM6597	2	+				DSMZ
Bacteroides uniformis CL03T12C37			NT5066	997890.6	HM-716	2	+				Nassos Typas

**Strain**	**Abbrev.**	**Relevant genotype ***	**Internal ID**	**PATRIC ID**	**Collection ID**	**Hazard group****	**100-strain screen**	**50-strain community**	***K. pneumoniae* 10 best-ranked**	***S.* Typhimurium 10 best-ranked**	**Source**
Bacteroides xylanisolvens XB1A			B12	657309.4	DSM18836	1	+	+			DSMZ
Bacteroides xylanisolvens CL03T12C04			NT5064	997892.50	DSM2079	2	+				Nassos Typas
Bifidobacterium adolescentis			NT5022	367928.6	DSM20083	1	+	+			Nassos Typas
Bifidobacterium animalis	*B. anim*		A2	555970.3	DSM10140	1	+	+	+	+	DSMZ
Bifidobacterium animalis subsp. lactis Bi-04			NT5043	580050.3		1	+				Nassos Typas
Bifidobacterium animalis subsp. lactis Bi-07			NT5044	742729.3	DGCC290 8	1	+	+			Nassos Typas
Bifidobacterium bifidum			A1	500634.6	DSM20456	1	+	+			DSMZ
Bifidobacterium breve	*B. brev*		A3	518634.19	DSM20213	1	+	+	+		DSMZ
Bifidobacterium longum subsp. infantis	*B. long. inf*		A4	391904.8	DSM20088	1	+	+	+		DSMZ

Strain	Abbrev.	Relevant genotype *	Internal ID	PATRIC ID	Collection ID	Hazard group**	100-strain screen	50-strain community	*K. pneumoniae* 10 best-ranked	*S.* Typhimurium 10 best-ranked	Source
Bifidobacterium longum subsp. longum	*B. long*. long		A5	565042.3	DSM20219	1	+	+		+	DSMZ
Blautia hansenii			NT5005	537007.6	DSM20583	1	+	+			Nassos Typas
Blautia hydrogenotrophica			F4	476272.21	DSM10507	1	+	+			DSMZ
Christensenella minuta			F7	626937.8	DSM22607	1	+	+			DSMZ
Clostridium difficile 630			NT23006	272563.8	DSM27543	2	+				Nassos Typas
Clostridium leptum			F12	428125.8	DSM753	1	+	+			DSMZ
Clostridium perfringens			NT5031	195103.10	DSM756	2	+				Nassos Typas
Clostridium perfringens			NT5032	451754.18	DSM11782	2	+				Nassos Typas
Collinsella aerofaciens			A6	411903.6	DSM3979	2	+				DSMZ

Strain	Abbrev.	Relevant genotype *	Internal ID	PATRIC ID	Collection ID	Hazard group**	100-strain screen	50-strain community	*K. pneumoniae* 10 best-ranked	*S.* Typhimurium 10 best-ranked	Source
Coprococcus comes			NT5048	470146.3	ATCC27758	1	+	+			Nassos Typas
Dorea formicigenerans			NT5076	411461.20	DSM3992	1	+	+			Nassos Typas
Dorea longicatena			F13	411462.6	DSM13814	1	+	+			DSMZ
Eggerthella lenta			NT5024	479437.5	DSM2243	2	+				Nassos Typas
Enterocloster bolteae			NT5026	208479.10	DSM15670	1	+	+			Nassos Typas
Erysipelatoclostridium ramosum			NT5006	445974.19	DSM1402	2	+				Nassos Typas
AMR *Escherichia coli*		Wildtype (Ampicillin)	19Y000018		-	2					Nottingham University Hospital Pathogen Bank
Escherichia coli ED1a			NT5078	585397.9		1	+				Nassos Typas
Escherichia coli HS			e-OPC-323	331112.6		1	+				(*34*)

**Strain**	**Abbrev.**	**Relevant genotype ***	**Internal ID**	**PATRIC ID**	**Collection ID**	**Hazard group****	**100-strain screen**	**50-strain community**	***K. pneumoniae*** **10 best-ranked**	***S.*** **Typhimurium 10 best-ranked**	**Source**
Escherichia coli IAI1	*E. coli*		NT5077	585034.5		1	+	+	+	+	Nassos Typas
Escherichia coli IAI1	*E. coli* Δ*gatABC*	Δ*gatABC*	eOPC-364			1					This study
Escherichia coli JKE201	+pOPC-231		eOPC-362			1					This study
Escherichia coli JKe201	+pOPC-232		eOPC-363			1					This study
Escherichia coli MG1655			e-OPC-292	511145.12		1	+				(*35*)
Escherichia coli Z1269			Z1269		-	1					(*33*)
Escherichia coli Z1331			Z1331		-	1					(*33*)
Eubacterium rectale			NT5009	657318.12	DSM17629	1	+	+			Nassos Typas
Eubacterium siraeum			NT5040	428128.19	DSM15702	1	+	+			Nassos Typas
Faecalibacterium prausnitzii			F25	411483.3	DSM17677	1		+			DSMZ

**Strain**	**Abbrev.**	**Relevant genotype***	**Internal ID**	**PATRIC ID**	**Collection ID**	**Hazard group****	**100-strain screen**	**50-strain community**	***K. pneumoniae* 10 best-ranked**	***S.* Typhimurium 10 best-ranked**	**Source**
Fusobacterium nucleatum CTI-01			NT24006	1204474.3		2	+				Nassos Typas
Fusobacterium nucleatum MJR7757B			NT24015	851.8		2	+				Nassos Typas
Fusobacterium nucleatum subsp. nucleatum			NT5025	190304.8	DSM15643	2	+				Nassos Typas
Fusobacterium nucleatum subsp. vincentii			NT24005	155615.5	DSM19508	2	+				Nassos Typas
Fusobacterium nucleatum subsp. vincentii			NT5030	209882.4	DSM19507	2	+				Nassos Typas
Fusobacterium periodonticum 1_A_54/D10			NT24011	546275.3	ATCC33693	1	+	+			Nassos Typas
Fusobacterium periodonticum 2_1_31			NT24012	469599.3		2	+				Nassos Typas
Gemella morbillorum			NT24013	562982.3		2	+				Nassos Typas
Holdemanella biformis	*H. bifo*		F15	518637.5	DSM3989	1	+	+	+		DSMZ

**Strain**	**Abbrev.**	**Relevant genotype***	**Internal ID**	**PATRIC ID**	**Collection ID**	**Hazard group****	**100-strain screen**	**50-strain community**	***K. pneumoniae* 10 best-ranked**	***S.* Typhimurium 10 best-ranked**	**Source**
Intestinibacter bartletti			NT5086	261299.118	DSM16795	1	+				Nassos Typas
Klebsiella pneumoniae			K1	272620.9	ATCC 700721	2	+				Modernising Medical Microbiology, Nuffield Department of Medicine
Klebsiella pneumoniae subsp. pneumoniae		Wildtype (Carbenicillin)	bFS-26	1162296.3	DSM 30104	2	+				DSMZ
Klebsiella pneumoniae subsp. pneumoniae		+ pRSJ-p_{nptII}::ilux	bFS-29		DSM 30104	2					This study
Klebsiella pneumoniae subsp. pneumoniae		+pBC11	bFS-34		DSM 30104	2					This study
Lachnoclostridium symbiosum WAL-14163			NT24007	742740.3		2	+				Nassos Typas

**Strain**	**Abbrev.**	**Relevant genotype ***	**Internal ID**	**PATRIC ID**	**Collection ID**	**Hazard group****	**100-strain screen**	**50-strain community**	***K. pneumoniae* 10 best-ranked**	***S.* Typhimurium 10 best-ranked**	**Source**
Lachnoclostridium symbiosum WAL-14673			NT24014	742741.3		2	+				Nassos Typas
Lachnoclostridium hylemonae			NT27002	553973.19	DSM15053	1	+	+			Nassos Typas
Lachnoclostridium scindens			F9	411468.41	DSM5676	1	+	+			DSMZ
Lachnoclostridium symbiosum			F8	411472.5	DSM934	2	+				DSMZ
Lacrimispora saccharolytica	*L. sacc*		NT5037	610130.3	DSM2544	1	+	+		+	Nassos Typas
Lacticaseibacillus casei			F16	219334.4	DSM20011	1	+	+			DSMZ
Lacticaseibacillus paracasei			NT5042	1226298.3	ATCCSD5275	1	+	+			Nassos Typas
Lactiplantibacillus plantarum JDM1	*L. plan*		F14	644042.3	-	1	+	+	+		Department of Food and Nutritional Sciences, University of Reading

**Strain**	**Abbrev.**	**Relevant genotype ***	**Internal ID**	**PATRIC ID**	**Collection ID**	**Hazard group****	**100-strain screen**	**50-strain community**	***K. pneumoniae* 10 best-ranked**	***S.* Typhimurium 10 best-ranked**	**Source**
Lactobacillus acidophilus	*L. acid*		NT5041	272621.13	ATCC700936	1	+	+	+	+	Nassos Typas
Lactobacillus delbrueckii subsp. Delbrueckii	*L. delb*		NT14075	1423823.4	DSM20074	1	+	+		+	Nassos Typas
Lactobacillus gasseri			F18	324831.13	DSM20243	1	+	+			DSMZ
Ligilactobacillus ruminis	*L. rumi*		F17	1423798.5	DSM20403	1	+	+	+		DSMZ
Ligilactobacillus salivarius	*L. sali*		NT14072	1423799.3	DSM20555	1	+	+	+	+	Nassos Typas
Limosilactobacillus fermentum	*L. ferm*		NT14076	1613.547	DSM20052	1	+	+	+		Nassos Typas
Mediterraneibacter gnavus			NT5046	411470.47	ATCC29149	1	+	+			Nassos Typas
Mediterraneibacter torques	*M. torq*		NT5047	411460.6	ATCC27756	1	+	+		+	Nassos Typas
Odoribacter splanchnicus			NT5081	709991.142	DSM20712	2	+				Nassos Typas

**Strain**	**Abbrev.**	**Relevant genotype***	**Internal ID**	**PATRIC ID**	**Collection ID**	**Hazard group****	**100-strain screen**	**50-strain community**	***K. pneumoniae* 10 best-ranked**	***S.* Typhimurium 10 best-ranked**	**Source**
Parabacteroides distasonis			NT5074	435591.48	DSM20701	2	+				Nassos Typas
Parabacteroides merdae			NT5071	411477.88	DSM19495	1	+				Nassos Typas
Peptostreptococcus stomatis			NT24002	596315.3	DSM17678	2	+				Nassos Typas
Phocaeicola coprocola			B3	470145.69	DSM17136	1	+	+			DSMZ
Phocaeicola dorei			NT5049	357276.1035	DSM17855	1	+	+			Nassos Typas
Phocaeicola massiliensis			B5	1121098.3	DSM17679	1	+	+			DSMZ
Phocaeicola vulgatus	*P. vulg*		B11	435590.9	DSM1447	1	+	+		+	DSMZ
Phocaeicola vulgatus CL09T03C04			NT5056	997891.3		1	+				Nassos Typas
Prevotella buccae			B14	873513.3	DSM19025	2	+				DSMZ

**Strain**	**Abbrev.**	**Relevant genotype ***	**Internal ID**	**PATRIC ID**	**Collection ID**	**Hazard group****	**100-strain screen**	**50-strain community**	***K. pneumoniae* 10 best-ranked**	***S.* Typhimurium 10 best-ranked**	**Source**
Prevotella copri			B15	537011.439	DSM18205	1	+	+			DSMZ
Roseburia faecis			F24	301302.4	DSM16840	1	+	+			DSMZ
Roseburia hominis			F22	585394.18	DSM16839	1	+	+			DSMZ
Roseburia intestinalis			NT5011	536231.75	DSM14610	1	+	+			Nassos Typas
Roseburia inulinivorans			NT5012	622312.48	DSM16841	1	+	+			Nassos Typas
Salmonella enterica Typhimurium SL1344		Wildtype (Streptomycin)	SB300	216597.6	DSM24522	2	+				(*58*)
Salmonella enterica Typhimurium SL1344		+ pRSJ-p_{nptII}::ilux	sOPC-406		DSM24522	2					This study
Salmonella enterica Typhimurium SL1344		+pBC11	sOPC-404		DSM24522	2					This study

**Strain**	**Abbrev.**	**Relevant genotype ***	**Internal ID**	**PATRIC ID**	**Collection ID**	**Hazard group****	**100-strain screen**	**50-strain community**	***K. pneumoniae* 10 best-ranked**	***S.* Typhimurium 10 best-ranked**	**Source**
Salmonella enterica Typhimurium SL1344		Avirulent	M2702		DSM24522	2					(*72*)
Salmonella enterica Typhimurium SL1344		*hisG* prototroph (Streptomycin)	EB199		DSM24522	2					This study
Salmonella enterica Typhimurium SL1344		Δ*gatABC* (Streptomycin)	sOPC-463		DSM24522	2					This study
Salmonella enterica Typhimurium SL1344		Δ*gatABC* +pBC11	EB149		DSM24522	2					This study
Staphylococcus epidermis			bOPC-105	176280.85	DSM1798	2	+				ATCC****
Streptococcus parasanguinis			NT5072	760570.3	DSM6778	2	+				Nassos Typas
Streptococcus salivarius			NT5038	1304.1829	DSM20560	2	+				Nassos Typas

Strain	Abbrev.	Relevant genotype *	Internal ID	PATRIC ID	Collection ID	Hazard group**	100-strain screen	50-strain community	*K. pneumoniae* 10 best-ranked	*S.* Typhimurium 10 best-ranked	Source
Veillonella parvula			NT5017	479436.6	DSM2008	2	+				Nassos Typas

Table S2.

Plasmids used in this study. *Relevant resistances only. Streptomycin= >50 ug/mL; carbenicillin=>50µg/mL; ampicillin=>100µg/mL; kanamycin=>50µg/mL; tetracycline=>50µg/mL.

**Plasmid name**	**Relevant genotype**	**Resistance***	**Source**
pRSJ-p_{nptII}::ilux	*lux*CDBAE-*frp* expression	Tetracycline	(*62*)
pBC11	YPet expression	Kanamycin	(*65*)
pOPC-231	SV-*aphT-tetR-sceI-sacB-S*Tm-*Δ*gatABC	Kanamycin	This study
pOPC-232	SV-*aphT-tetR-sceI-sacB-Ec*IAI-*Δ*gatABC	Kanamycin	This study

Table S3.

Primers used in this study.

Primer name	Sequence	Purpose	Source
oOPC-953	AGAGTTTGATCCTGGCTCAG	16S sequencing (27F)	(*73*)
oOPC-954	TACGGYTACCTTGTTACGACTT	16S sequencing (1492R)	(*73*)
g-Bifid-F	5′-CTCCTGGAAACGGGTGG-3′	16S sequencing for Bifidobacteria	(*74*)
g-Bifid-R	5′-GGTGTTCTTCCCGATATCTACA-3′		(*74*)
oOPC-975	CCCAGTCTCGAGGTCGACGGTATCGATAAGCTTGA TATCGAATTCaaatcgcgtttcgtgaatcagg	amplify 700 upstream *gatABC* for deletion - STm SL1344	This study
oOPC-976	taaaattaagaggcgatttgaaatagttggctcataaattctccattattcagg		This study
oOPC-977	aatttatgagccaactatttcaaatcgcctcttaattttaggggag	amplify 700 downstream *gatABC* for deletion - *S*Tm	This study
oOPC-978	CTGGAGCTCCACCGCGGTGGCGGCCGCTCTAGAAC TAGTGGATCCttcccggctattacaggtatgcgttgcgc		This study
oOPC-979	agtcgatgctgcacgtacgc	check *gatABC* deletion - *S*Tm	This study
oOPC-980	atgtcggacaacgcggtctg		This study
oOPC-981	CCCAGTCTCGAGGTCGACGGTATCGATAAGCTTGA TATCGAATTCctactgttaaatgtttgcatcgcacc	amplify 700 upstream *gatABC* for deletion - *E.coli* IAI1	This study
oOPC-982	ggtatatgactaacctgtttgttctcgcagaataatttttacctgaggg		This study
oOPC-983	aaaaattattctgcgagaacaaacaggttagtcatataccgtccttattccg	amplify 700 downstream *gatABC* for deletion - *E.coli* IAI1	This study
oOPC-984	CTGGAGCTCCACCGCGGTGGCGGCCGCTCTAGAAC TAGTGGATCCtttacgtacgcatcaaaaggcctttattgcc		This study
oOPC-985	aaacgctctgcatttgcggc	check *gatABC* deletion - *E.coli* IAI1	This study
oOPC-986	cccatgttgaagatgccgcc		This study

Table S4.

Species compositions of communities used for *in vitro* experiments with *K. pneumoniae* DSM 30104. *Communities that were additionally selected to contain *E. coli* IAI1, but otherwise were selected at random.

**Community ID**	**Community members**	**No. replicates**	**Median day 2 pathogen density (cells/mL)**
No commensals	N/A	15	1.84E+09
KTS1	A3	5	9.45E+08
KTS2	F15	3	1.11E+09
KTS3	F17	3	1.19E+09
KTS4	A4	3	1.29E+09
KTS5	NT5077	3	1.10E+08
KTS6	NT5041	3	1.12E+09
KTS7	A2	3	1.28E+09
KTS8	F14	3	1.21E+09
KTS9	NT14076	3	1.68E+09
KTS10	NT14072	3	1.53E+09
KTD1	A4, NT5041	3	1.33E+09
KTD2	NT5041, F14	3	1.29E+09
KTD3	A3, NT5077	4	4.58E+06
KTD4	F15, F14	3	1.40E+09
KTD5	F15, A2	3	1.83E+09
KTD6	A2, F14	3	1.70E+09
KTD7	F17, NT14072	3	1.59E+09
KTD8	NT5077, NT14076	4	1.28E+08
KTD9	A3, F14	3	5.96E+08
KTD10*	NT5077, F14	3	1.82E+07
KTD11*	F15, NT5077	3	1.09E+08
KTD12*	F17, NT5077	3	3.42E+07

**Community ID**	**Community members**	**No. replicates**	**Median day 2 pathogen density (cells/mL)**
KTD13*	A4, NT5077	3	7.44E+07
KTD14*	NT5077, NT5041	3	1.55E+07
KTD15*	NT5077, A2	3	3.84E+07
KTD16*	NT5077, NT14072	3	2.90E+07
KTD17*	NT5041, A2	3	1.81E+09
KTT1	A3, F15, NT5041	3	3.30E+08
KTT2	A3, F15, A2	3	7.44E+08
KTT3	A2, NT14076, NT14072	3	6.56E+08
KTT4	F17, NT5077, NT14072	3	1.12E+07
KTT5	NT5041, NT14076, NT14072	3	7.11E+08
KTT6	F17, NT5077, A2	3	2.52E+07
KTT7	F17, A4, NT14076	3	1.07E+09
KTT8	A3, F15, F17	4	5.03E+08
KTT9	A3, NT5041, A2	3	7.59E+08
KTT10	A3, A4, NT5041	3	6.13E+08
KTT11	A2, F14, NT14072	3	1.19E+09
KTT12	A4, NT5041, F14	3	9.36E+08
KTT13	F15, F14, NT14072	3	1.13E+09
KTT14	A3, NT5077, F14	3	3.81E+05
KTT15	A3, F17, A2	3	5.07E+08
KTT16	NT5077, F14, NT14072	3	1.72E+07
KTT17	NT5077, NT14076, NT14072	3	1.43E+07
KTT23*	A3, F15, NT5077	2	8.73E+05
KTT24*	A4, NT5077, NT14072	3	1.29E+07
KTT25*	A3, NT5077, A2	3	6.53E+05
KTT26*	NT5077, NT5041, NT14072	3	6.20E+06
KTP1	A3, F15, F17, NT5041, NT14076	4	4.77E+08
KTP2	A3, F15, NT5041, A2, NT14072	4	2.98E+08

Community ID	Community members	No. replicates	Median day 2 pathogen density (cells/mL)
KTP3	NT5077, A2, F14, NT14076, NT14072	4	1.66E+07
KTP4	F17, NT5077, NT5041, A2, NT14072	4	2.42E+07
KTP5	A3, F15, NT5041, NT14076, NT14072	4	3.63E+08
KTP6	A3, F15, F17, A4, A2	4	5.33E+08
KTP7	F17, A4, NT5077, A2, NT14076	4	2.63E+07
KTP8	A3, F15, F17, NT5077, NT5041	4	1.25E+06
KTP9	A3, A4, NT5041, A2, F14	4	1.85E+08
KTP10	A3, A4, NT5041, F14, NT14072	4	1.99E+08
KTP11	F15, NT5041, A2, F14, NT14072	4	1.19E+09
KTP12	F17, NT5041, A2, F14, NT14076	4	9.19E+08
KTP13	F15, A4, F14, NT14076, NT14072	4	9.89E+08
KTP14	A3, A4, A2, F14, NT14076	4	6.60E+08
KTP15	F17, A4, F14, NT14076, NT14072	4	1.28E+09
KTP16	A3, F17, A4, F14, NT14072	4	2.95E+08
KTP17	A3, A4, A2, NT14076, NT14072	4	2.67E+08
KTP18*	F15, NT5077, NT5041, A2, F14	3	2.72E+06
KTP19*	F17, A4, NT5077, F14, NT14076	3	1.28E+07
KTP20*	A3, NT5077, NT5041, A2, NT14076	3	2.82E+05
KTP21*	A3, F15, NT5077, NT5041, F14	3	3.26E+05
KTP22*	A3, F17, NT5077, NT5041, F14	3	3.08E+05
KALLTOP	A3, F15, F17, A4, NT5077, NT5041, A2, F14, NT14076, NT14072	11	6.17E+05

Table S5.

Species compositions of communities used for *in vitro* experiments with *S. enterica* serovar Typhimurium SL1344. *Communities that were additionally selected to contain *E. coli* IAI1, but otherwise were selected at random.

**Community ID**	**Community members**	**No. replicates**	**Median day 2 pathogen density (cells/mL)**
No commensals	N/A	11	1.38E+09
STS1	A2	3	7.64E+08
STS2	NT5037	3	6.29E+08
STS3	NT14075	3	8.71E+08
STS4	F3	3	9.55E+08
STS5	NT5041	3	9.26E+08
STS6	A5	3	8.69E+08
STS7	NT5077	5	4.46E+08
STS8	B11	4	8.14E+08
STS9	NT5047	3	1.18E+09
STS10	NT14072	3	1.08E+09
STD1	NT5037, B11	3	6.04E+08
STD2	NT5077, NT5047	3	3.60E+08
STD3	NT5041, B11	3	7.16E+08
STD4	A5, NT5047	3	9.19E+08
STD5	NT14075, NT5077	3	3.98E+08
STD6	NT5037, NT14072	3	6.30E+08
STD7	B11, NT5047	3	7.64E+08
STD8	NT14075, NT14072	3	9.23E+08
STD9	NT5037, NT5047	3	7.74E+08
STD10	NT14075, NT5047	3	9.25E+08
STD11	NT14075, NT5041	3	9.62E+08
STD12	NT5041, NT14072	3	9.98E+08

Community ID	Community members	No. replicates	Median day 2 pathogen density (cells/mL)
STD13	A2, NT14072	3	1.06E+09
STD14	A2, NT5047	3	8.40E+08
STD15	B11, NT14072	3	7.79E+08
STD16	NT14075, F3	3	1.07E+09
STD17*	A2, NT5077	3	3.36E+08
STD18*	NT5037, NT5077	4	5.73E+07
STD19*	F3, NT5077	3	3.23E+08
STD20*	NT5041, NT5077	3	3.11E+08
STD21*	A5, NT5077	3	3.52E+08
STD22*	NT5077, B11	7	3.94E+07
STD23*	NT5077, NT14072	3	4.14E+08
STT1	A2, NT14075, A5	3	9.50E+08
STT2	NT14075, F3, B11	3	1.00E+09
STT3	F3, NT5041, NT5077	3	5.51E+08
STT4	A2, A5, NT14072	3	1.15E+09
STT5	A2, NT5037, A5	3	9.38E+08
STT6	NT5037, NT5077, NT14072	3	1.25E+08
STT7	F3, NT5041, A5	3	1.26E+09
STT8	NT5037, A5, NT5047	3	1.05E+09
STT9	NT5037, F3, NT5077	3	1.44E+08
STT10	NT5037, A5, NT14072	3	9.17E+08
STT11	NT14075, B11, NT5047	3	9.20E+08
STT12	A2, NT14075, B11	3	1.10E+09
STT13	A5, B11, NT14072	3	9.89E+08
STT14	NT5041, NT5047, NT14072	3	1.26E+09
STT15	A5, B11, NT5047	3	1.25E+09
STT16	A2, F3, B11	3	1.22E+09
STT17*	NT5037, NT5077, B11	5	2.23E+07

Community ID	Community members	No. replicates	Median day 2 pathogen density (cells/mL)
STT18*	A2, NT14075, NT5077	3	3.75E+08
STT19*	NT5041, NT5077, NT5047	3	3.80E+08
STT20*	NT5037, NT5077, NT5047	3	6.17E+07
STT21*	F3, NT5077, NT5047	3	3.79E+08
STT22*	NT14075, NT5077, B11	3	3.87E+07
STP1	NT14075, F3, NT5041, NT5047, NT14072	4	1.19E+09
STP2	A2, NT5037, NT14075, NT5077, NT14072	4	1.65E+08
STP3	NT14075, F3, A5, B11, NT14072	4	9.01E+08
STP4	A2, F3, A5, B11, NT5047	4	1.05E+09
STP5	F3, NT5041, A5, NT5077, B11	4	4.53E+07
STP6	NT5037, F3, B11, NT5047, NT14072	4	6.69E+08
STP7	NT14075, F3, NT5041, A5, NT5047	4	1.09E+09
STP8	A2, NT5037, F3, NT5041, NT5047	4	1.07E+09
STP9	NT5037, NT14075, A5, B11, NT14072	4	9.40E+08
STP10	A2, F3, NT5041, A5, NT5047	4	1.40E+09
STP11	A2, NT5037, A5, NT5077, B11	4	2.55E+07
STP12	NT5037, A5, NT5077, NT5047, NT14072	4	1.46E+08
STP13	A2, NT14075, A5, B11, NT14072	4	8.71E+08
STP14	F3, NT5041, NT5077, NT5047, NT14072	4	4.59E+08
STP15	A2, NT5037, NT14075, F3, A5	4	1.06E+09
STP16	A2, NT14075, F3, A5, NT14072	4	1.19E+09
STP17*	NT5041, A5, NT5077, B11, NT14072	3	2.01E+07
STP18*	NT5037, NT5041, A5, NT5077, B11	3	9.83E+06
STP19*	NT14075, F3, A5, NT5077, NT14072	3	4.38E+08
STP20*	A2, F3, A5, NT5077, B11	3	1.83E+07
SALLTOP	A2, NT5037, NT14075, F3, NT5041, A5, NT5077, B11, NT5047, NT14072	11	1.76E+07

Table S6.

Species compositions of communities used for gnotobiotic mouse experiments.

**Pathogen gavaged**	**No. symbionts in community**	**Community members**	***E. coli* IAI1 present**	**No. replicates**	**Median pathogen fecal density 24 hours p.i. (CFU/g)**
K. pneumoniae	0	N/A	No	7	4.0375e+09
K. pneumoniae	1	NT5077	Yes	7	2.28846e+08
K. pneumoniae	5	KTP8 (see Table S4)	Yes	8	4.35148e+07
K. pneumoniae	10	KALLTOP (see Table S4)	Yes	7	4.07019e+07
K. pneumoniae	50	See Table S1	Yes	7	134667
K. pneumoniae	9	KALLTOP minus NT5077	No	7	4.4e+08
K. pneumoniae	49	50 minus NT5077	No	7	2.32e+07
S. Typhimurium	0	N/A	No	7	7.89e+08
S. Typhimurium	1	NT5077	Yes	7	9.01408e+07
S. Typhimurium	5	STP11 (see Table S5)	Yes	8	5.68115e+07
S. Typhimurium	10	SALLTOP (see Table S5)	Yes	7	4.1e+07
S. Typhimurium	50	See Table S1	Yes	7	521000
S. Typhimurium	9	SALLTOP minus NT5077	No	8	6.91e+08
S. Typhimurium	49	50 minus NT5077	No	7	2.15e+07

Table S7.

Species compositions of communities used for prediction experiments with the AMR *E. coli* strain 19Y000018.

**Community ID**	**Community members**	**No. replicates**	**Median day 2 pathogen density (CFU/mL)**
No commensals	N/A	5	4.96E+08
Best 2 Biolog	NT5077, NT5037	5	1.00E+07
Worst 2 Biolog	NT5077, A2	5	3.12E+08
Best 3 Biolog	NT5077, B11, NT5037	5	1.56E+06
Worst 3 Biolog	NT5077, F17, NT5041	5	2.64E+08
Best 5 Biolog	NT5077, NT5037, NT14075, NT5041, B11	5	2.48E+06
Worst 5 Biolog	NT5077, NT14075, A5, A3, F17	5	4.64E+06
Best 2 protein family	NT5077, NT5026	5	1.80E+07
Worst 2 protein family	NT5077, F3	5	2.48E+08
Best 3 protein family	NT5077, NT5026, F2	5	1.60E+07
Worst 3 protein family	NT5077, A2, NT5044	5	2.24E+08
Best 5 protein family	NT5077, NT5026, F2, NT5049, NT24011	5	2.80E+06
Worst 5 protein family	NT5077, NT14072, NT5041, F24, NT5040	5	8.16E+06
Best 10 protein family	NT5077, F2, NT14076, NT5026, NT5049, NT5047, F25, NT24011, NT5037, NT5012	5	1.60E+06
Worst 10 protein family	NT5077, F17, NT5041, NT5076, NT5048, A1, F15, A2, NT5009, NT5044	5	3.40E+06
Best 5 protein family #2	NT5077, NT5026, NT24011, NT5037, NT5052	5	4.00E+06

Community ID	Community members	No. replicates	Median day 2 pathogen density (CFU/mL)
Best 5 protein family #3	NT5077, NT5026, NT24011, NT5037, NT5049	5	2.02E+06
Best 5 protein family #4	NT5077, F2, NT24011, NT5037, NT5052	5	5.52E+06
Best 5 protein family #5	NT5077, F2, NT5026, F16, NT5049	5	2.48E+06
Worst 5 protein family #2	NT5077, F17, F15, A2, NT5044	5	3.00E+08
Worst 5 protein family #3	NT5077, NT5048, F15, A2, NT5044	5	2.32E+08
Worst 5 protein family #4	NT5077, NT14072, NT5040, A2, NT5044	5	3.20E+08
Worst 5 protein family #5	NT5077, F17, A1, A2, NT5044	5	2.80E+08

References and Notes

1. M. T. Sorbara, E. G. Pamer, Interbacterial mechanisms of colonization resistance and the strategies pathogens use to overcome them. *Mucosal Immunol.* **12**, 1–9 (2019). doi:10.1038/s41385-018-0053-0 Medline

2. A. Jacobson, L. Lam, M. Rajendram, F. Tamburini, J. Honeycutt, T. Pham, W. Van Treuren, K. Pruss, S. R. Stabler, K. Lugo, D. M. Bouley, J. G. Vilches-Moure, M. Smith, J. L. Sonnenburg, A. S. Bhatt, K. C. Huang, D. Monack, A Gut Commensal-Produced Metabolite Mediates Colonization Resistance to Salmonella Infection. *Cell Host Microbe* **24**, 296–307.e7 (2018). doi:10.1016/j.chom.2018.07.002 Medline

3. E. M. Velazquez, H. Nguyen, K. T. Heasley, C. H. Saechao, L. M. Gil, A. W. L. Rogers, B. M. Miller, M. R. Rolston, C. A. Lopez, Y. Litvak, M. J. Liou, F. Faber, D. N. Bronner, C. R. Tiffany, M. X. Byndloss, A. J. Byndloss, A. J. Bäumler, Endogenous Enterobacteriaceae underlie variation in susceptibility to Salmonella infection. *Nat. Microbiol.* **4**, 1057–1064 (2019). doi:10.1038/s41564-019-0407-8 Medline

4. S. Y. Wotzka, M. Kreuzer, L. Maier, M. Arnoldini, B. D. Nguyen, A. O. Brachmann, D. L. Berthold, M. Zünd, A. Hausmann, E. Bakkeren, D. Hoces, E. Gül, M. Beutler, T. Dolowschiak, M. Zimmermann, T. Fuhrer, K. Moor, U. Sauer, A. Typas, J. Piel, M. Diard, A. J. Macpherson, B. Stecher, S. Sunagawa, E. Slack, W.-D. Hardt, Escherichia coli limits Salmonella Typhimurium infections after diet shifts and fat-mediated microbiota perturbation in mice. *Nat. Microbiol.* **4**, 2164–2174 (2019). doi:10.1038/s41564-019-0568-5 Medline

5. R. P. Sequeira, J. A. K. McDonald, J. R. Marchesi, T. B. Clarke, Commensal Bacteroidetes protect against Klebsiella pneumoniae colonization and transmission through IL-36 signalling. *Nat. Microbiol.* **5**, 304–313 (2020). doi:10.1038/s41564-019-0640-1 Medline

6. C. G. Buffie, V. Bucci, R. R. Stein, P. T. McKenney, L. Ling, A. Gobourne, D. No, H. Liu, M. Kinnebrew, A. Viale, E. Littmann, M. R. M. van den Brink, R. R. Jenq, Y. Taur, C. Sander, J. R. Cross, N. C. Toussaint, J. B. Xavier, E. G. Pamer, Precision microbiome reconstitution restores bile acid mediated resistance to Clostridium difficile. *Nature* **517**, 205–208 (2015). doi:10.1038/nature13828 Medline

7. S. G. Kim, S. Becattini, T. U. Moody, P. V. Shliaha, E. R. Littmann, R. Seok, M. Gjonbalaj, V. Eaton, E. Fontana, L. Amoretti, R. Wright, S. Caballero, Z. X. Wang, H.-J. Jung, S. M. Morjaria, I. M. Leiner, W. Qin, R. J. J. F. Ramos, J. R. Cross, S. Narushima, K. Honda, J. U. Peled, R. C. Hendrickson, Y. Taur, M. R. M. van den Brink, E. G. Pamer, Microbiota-derived lantibiotic restores resistance against vancomycin-resistant Enterococcus. *Nature* **572**, 665–669 (2019). doi:10.1038/s41586-019-1501-z Medline

8. M. X. Byndloss, E. E. Olsan, F. Rivera-Chávez, C. R. Tiffany, S. A. Cevallos, K. L. Lokken, T. P. Torres, A. J. Byndloss, F. Faber, Y. Gao, Y. Litvak, C. A. Lopez, G. Xu, E. Napoli, C. Giulivi, R. M. Tsolis, A. Revzin, C. B. Lebrilla, A. J. Bäumler, Microbiota-activated PPAR-γ signaling inhibits dysbiotic Enterobacteriaceae expansion. *Science* **357**, 570–575 (2017). doi:10.1126/science.aam9949 Medline

9. R. A. Oliveira, K. M. Ng, M. B. Correia, V. Cabral, H. Shi, J. L. Sonnenburg, K. C. Huang, K. B. Xavier, Klebsiella michiganensis transmission enhances resistance to

Enterobacteriaceae gut invasion by nutrition competition. *Nat. Microbiol.* **5**, 630–641 (2020). doi:10.1038/s41564-019-0658-4 Medline

10. G. Caballero-Flores, J. M. Pickard, G. Núñez, Microbiota-mediated colonization resistance: Mechanisms and regulation. *Nat. Rev. Microbiol.* **21**, 347–360 (2023). doi:10.1038/s41579-022-00833-7 Medline

11. E. T. Granato, T. A. Meiller-Legrand, K. R. Foster, The Evolution and Ecology of Bacterial Warfare. *Curr. Biol.* **29**, R521–R537 (2019). doi:10.1016/j.cub.2019.04.024 Medline

12. C. Eberl, A. S. Weiss, L. M. Jochum, A. C. Durai Raj, D. Ring, S. Hussain, S. Herp, C. Meng, K. Kleigrewe, M. Gigl, M. Basic, B. Stecher, E. coli enhance colonization resistance against Salmonella Typhimurium by competing for galactitol, a context-dependent limiting carbon source. *Cell Host Microbe* **29**, 1680–1692.e7 (2021). doi:10.1016/j.chom.2021.09.004 Medline

13. L. Osbelt, M. Wende, É. Almási, E. Derksen, U. Muthukumarasamy, T. R. Lesker, E. J. C. Galvez, M. C. Pils, E. Schalk, P. Chhatwal, J. Färber, M. Neumann-Schaal, T. Fischer, D. Schlüter, T. Strowig, Klebsiella oxytoca causes colonization resistance against multidrug-resistant K. pneumoniae in the gut via cooperative carbohydrate competition. *Cell Host Microbe* **29**, 1663–1679.e7 (2021). doi:10.1016/j.chom.2021.09.003 Medline

14. S. Caballero, S. Kim, R. A. Carter, I. M. Leiner, B. Sušac, L. Miller, G. J. Kim, L. Ling, E. G. Pamer, Cooperating Commensals Restore Colonization Resistance to Vancomycin-Resistant Enterococcus faecium. *Cell Host Microbe* **21**, 592–602.e4 (2017). doi:10.1016/j.chom.2017.04.002 Medline

15. S. Brugiroux, M. Beutler, C. Pfann, D. Garzetti, H.-J. Ruscheweyh, D. Ring, M. Diehl, S. Herp, Y. Lötscher, S. Hussain, B. Bunk, R. Pukall, D. H. Huson, P. C. Münch, A. C. McHardy, K. D. McCoy, A. J. Macpherson, A. Loy, T. Clavel, D. Berry, B. Stecher, Genome-guided design of a defined mouse microbiota that confers colonization resistance against Salmonella enterica serovar Typhimurium. *Nat. Microbiol.* **2**, 16215 (2016). doi:10.1038/nmicrobiol.2016.215 Medline

16. A. G. Cheng, P.-Y. Ho, A. Aranda-Díaz, S. Jain, F. B. Yu, X. Meng, M. Wang, M. Iakiviak, K. Nagashima, A. Zhao, P. Murugkar, A. Patil, K. Atabakhsh, A. Weakley, J. Yan, A. R. Brumbaugh, S. Higginbottom, A. Dimas, A. L. Shiver, A. Deutschbauer, N. Neff, J. L. Sonnenburg, K. C. Huang, M. A. Fischbach, Design, construction, and in vivo augmentation of a complex gut microbiome. *Cell* **185**, 3617–3636.e19 (2022). doi:10.1016/j.cell.2022.08.003 Medline

17. S. Widder, R. J. Allen, T. Pfeiffer, T. P. Curtis, C. Wiuf, W. T. Sloan, O. X. Cordero, S. P. Brown, B. Momeni, W. Shou, H. Kettle, H. J. Flint, A. F. Haas, B. Laroche, J.-U. Kreft, P. B. Rainey, S. Freilich, S. Schuster, K. Milferstedt, J. R. van der Meer, T. Großkopf, J. Huisman, A. Free, C. Picioreanu, C. Quince, I. Klapper, S. Labarthe, B. F. Smets, H. Wang, O. S. Soyer; Isaac Newton Institute Fellows, Challenges in microbial ecology: Building predictive understanding of community function and dynamics. *ISME J.* **10**, 2557–2568 (2016). doi:10.1038/ismej.2016.45 Medline

18. E. Tacconelli, E. Carrara, A. Savoldi, S. Harbarth, M. Mendelson, D. L. Monnet, C. Pulcini, G. Kahlmeter, J. Kluytmans, Y. Carmeli, M. Ouellette, K. Outterson, J. Patel, M. Cavaleri, E. M. Cox, C. R. Houchens, M. L. Grayson, P. Hansen, N. Singh, U.

Theuretzbacher, N. Magrini, A. O. Aboderin, S. S. Al-Abri, N. Awang Jalil, N. Benzonana, S. Bhattacharya, A. J. Brink, F. R. Burkert, O. Cars, G. Cornaglia, O. J. Dyar, A. W. Friedrich, A. C. Gales, S. Gandra, C. G. Giske, D. A. Goff, H. Goossens, T. Gottlieb, M. Guzman Blanco, W. Hryniewicz, D. Kattula, T. Jinks, S. S. Kanj, L. Kerr, M.-P. Kieny, Y. S. Kim, R. S. Kozlov, J. Labarca, R. Laxminarayan, K. Leder, L. Leibovici, G. Levy-Hara, J. Littman, S. Malhotra-Kumar, V. Manchanda, L. Moja, B. Ndoye, A. Pan, D. L. Paterson, M. Paul, H. Qiu, P. Ramon-Pardo, J. Rodríguez-Baño, M. Sanguinetti, S. Sengupta, M. Sharland, M. Si-Mehand, L. L. Silver, W. Song, M. Steinbakk, J. Thomsen, G. E. Thwaites, J. W. M. van der Meer, N. Van Kinh, S. Vega, M. V. Villegas, A. Wechsler-Fördös, H. F. L. Wertheim, E. Wesangula, N. Woodford, F. O. Yilmaz, A. Zorzet, WHO Pathogens Priority List Working Group, Discovery, research, and development of new antibiotics: The WHO priority list of antibiotic-resistant bacteria and tuberculosis. *Lancet Infect. Dis.* **18**, 318–327 (2018). doi:10.1016/S1473-3099(17)30753-3 Medline

19. B. Stecher, R. Robbiani, A. W. Walker, A. M. Westendorf, M. Barthel, M. Kremer, S. Chaffron, A. J. Macpherson, J. Buer, J. Parkhill, G. Dougan, C. von Mering, W.-D. Hardt, Salmonella enterica serovar typhimurium exploits inflammation to compete with the intestinal microbiota. *PLOS Biol.* **5**, e244 (2007). doi:10.1371/journal.pbio.0050244 Medline

20. S. E. Majowicz, J. Musto, E. Scallan, F. J. Angulo, M. Kirk, S. J. O'Brien, T. F. Jones, A. Fazil, R. M. Hoekstra, International Collaboration on Enteric Disease 'Burden of Illness' Studies, The global burden of nontyphoidal Salmonella gastroenteritis. *Clin. Infect. Dis.* **50**, 882–889 (2010). doi:10.1086/650733 Medline

21. C. L. Gorrie, M. Mirčeta, R. R. Wick, D. J. Edwards, N. R. Thomson, R. A. Strugnell, N. F. Pratt, J. S. Garlick, K. M. Watson, D. V. Pilcher, S. A. McGloughlin, D. W. Spelman, A. W. J. Jenney, K. E. Holt, Gastrointestinal Carriage Is a Major Reservoir of Klebsiella pneumoniae Infection in Intensive Care Patients. *Clin. Infect. Dis.* **65**, 208–215 (2017). doi:10.1093/cid/cix270 Medline

22. M. M. Mayfield, D. B. Stouffer, Higher-order interactions capture unexplained complexity in diverse communities. *Nat. Ecol. Evol.* **1**, 62 (2017). doi:10.1038/s41559-016-0062 Medline

23. Y. Litvak, A. J. Bäumler, The founder hypothesis: A basis for microbiota resistance, diversity in taxa carriage, and colonization resistance against pathogens. *PLOS Pathog.* **15**, e1007563 (2019). doi:10.1371/journal.ppat.1007563 Medline

24. K. Z. Coyte, S. Rakoff-Nahoum, Understanding Competition and Cooperation within the Mammalian Gut Microbiome. *Curr. Biol.* **29**, R538–R544 (2019). doi:10.1016/j.cub.2019.04.017 Medline

25. O. Manor, C. L. Dai, S. A. Kornilov, B. Smith, N. D. Price, J. C. Lovejoy, S. M. Gibbons, A. T. Magis, Health and disease markers correlate with gut microbiome composition across thousands of people. *Nat. Commun.* **11**, 5206 (2020). doi:10.1038/s41467-020-18871-1 Medline

26. C. A. Lozupone, J. I. Stombaugh, J. I. Gordon, J. K. Jansson, R. Knight, Diversity, stability and resilience of the human gut microbiota. *Nature* **489**, 220–230 (2012). doi:10.1038/nature11550 Medline

27. C. Tropini, E. L. Moss, B. D. Merrill, K. M. Ng, S. K. Higginbottom, E. P. Casavant, C. G. Gonzalez, B. Fremin, D. M. Bouley, J. E. Elias, A. S. Bhatt, K. C. Huang, J. L. Sonnenburg, Transient Osmotic Perturbation Causes Long-Term Alteration to the Gut Microbiota. *Cell* **173**, 1742–1754.e17 (2018). doi:10.1016/j.cell.2018.05.008 Medline

28. K. V. Johnson, P. W. Burnet, Microbiome: Should we diversify from diversity? *Gut Microbes* **7**, 455–458 (2016). doi:10.1080/19490976.2016.1241933 Medline

29. A. Baichman-Kass, T. Song, J. Friedman, Competitive interactions between culturable bacteria are highly non-additive. *eLife* **12**, e83398 (2023). doi:10.7554/eLife.83398 Medline

30. I. Billick, T. J. Case, Higher Order Interactions in Ecological Communities: What Are They and How Can They be Detected? *Ecology* **75**, 1529–1543 (1994). doi:10.2307/1939614

31. A. L. Gould, V. Zhang, L. Lamberti, E. W. Jones, B. Obadia, N. Korasidis, A. Gavryushkin, J. M. Carlson, N. Beerenwinkel, W. B. Ludington, Microbiome interactions shape host fitness. *Proc. Natl. Acad. Sci. U.S.A.* **115**, E11951–E11960 (2018). doi:10.1073/pnas.1809349115 Medline

32. W. B. Ludington, Higher-order microbiome interactions and how to find them. *Trends Microbiol.* **30**, 618–621 (2022). doi:10.1016/j.tim.2022.03.011 Medline

33. S. Y. Wotzka, M. Kreuzer, L. Maier, M. Zünd, M. Schlumberger, B. Nguyen, M. Fox, D. Pohl, H. Heinrich, G. Rogler, L. Biedermann, M. Scharl, S. Sunagawa, W.-D. Hardt, B. Misselwitz, Microbiota stability in healthy individuals after single-dose lactulose challenge-A randomized controlled study. *PLOS ONE* **13**, e0206214 (2018). doi:10.1371/journal.pone.0206214 Medline

34. S. B. Formal, G. J. Dammin, E. H. Labrec, H. Schneider, Experimental Shigella infections: Characteristics of a fatal infection produced in guinea pigs. *J. Bacteriol.* **75**, 604–610 (1958). doi:10.1128/jb.75.5.604-610.1958 Medline

35. F. R. Blattner, G. Plunkett 3rd, C. A. Bloch, N. T. Perna, V. Burland, M. Riley, J. Collado-Vides, J. D. Glasner, C. K. Rode, G. F. Mayhew, J. Gregor, N. W. Davis, H. A. Kirkpatrick, M. A. Goeden, D. J. Rose, B. Mau, Y. Shao, The complete genome sequence of Escherichia coli K-12. *Science* **277**, 1453–1462 (1997). doi:10.1126/science.277.5331.1453 Medline

36. M. Ackermann, B. Stecher, N. E. Freed, P. Songhet, W.-D. Hardt, M. Doebeli, Self-destructive cooperation mediated by phenotypic noise. *Nature* **454**, 987–990 (2008). doi:10.1038/nature07067 Medline

37. E. Gül, E. Bakkeren, G. Salazar, Y. Steiger, A. Abi Younes, M. Clerc, P. Christen, S. A. Fattinger, B. D. Nguyen, P. Kiefer, E. Slack, M. Ackermann, J. A. Vorholt, S. Sunagawa, M. Diard, W.-D. Hardt, The microbiota conditions a gut milieu that selects for wild-type Salmonella Typhimurium virulence. *PLOS Biol.* **21**, e3002253 (2023). doi:10.1371/journal.pbio.3002253 Medline

38. R. J. Gibbons, S. S. Socransky, B. Kapsimalis, Establishment of Human Indigenous Bacteria in Germ-Free Mice. *J. Bacteriol.* **88**, 1316–1323 (1964). doi:10.1128/jb.88.5.1316-1323.1964 Medline

39. R. Freter, H. Brickner, M. Botney, D. Cleven, A. Aranki, Mechanisms that control bacterial populations in continuous-flow culture models of mouse large intestinal flora. *Infect. Immun.* **39**, 676–685 (1983). doi:10.1128/iai.39.2.676-685.1983 Medline

40. A. Wagner, Competition for nutrients increases invasion resistance during assembly of microbial communities. *Mol. Ecol.* **31**, 4188–4203 (2022). doi:10.1111/mec.16565 Medline

41. C. J. L. Murray, K. S. Ikuta, F. Sharara, L. Swetschinski, G. Robles Aguilar, A. Gray, C. Han, C. Bisignano, P. Rao, E. Wool, S. C. Johnson, A. J. Browne, M. G. Chipeta, F. Fell, S. Hackett, G. Haines-Woodhouse, B. H. Kashef Hamadani, E. A. P. Kumaran, B. McManigal, S. Achalapong, R. Agarwal, S. Akech, S. Albertson, J. Amuasi, J. Andrews, A. Aravkin, E. Ashley, F.-X. Babin, F. Bailey, S. Baker, B. Basnyat, A. Bekker, R. Bender, J. A. Berkley, A. Bethou, J. Bielicki, S. Boonkasidecha, J. Bukosia, C. Carvalheiro, C. Castañeda-Orjuela, V. Chansamouth, S. Chaurasia, S. Chiurchiù, F. Chowdhury, R. Clotaire Donatien, A. J. Cook, B. Cooper, T. R. Cressey, E. Criollo-Mora, M. Cunningham, S. Darboe, N. P. J. Day, M. De Luca, K. Dokova, A. Dramowski, S. J. Dunachie, T. Duong Bich, T. Eckmanns, D. Eibach, A. Emami, N. Feasey, N. Fisher-Pearson, K. Forrest, C. Garcia, D. Garrett, P. Gastmeier, A. Z. Giref, R. C. Greer, V. Gupta, S. Haller, A. Haselbeck, S. I. Hay, M. Holm, S. Hopkins, Y. Hsia, K. C. Iregbu, J. Jacobs, D. Jarovsky, F. Javanmardi, A. W. J. Jenney, M. Khorana, S. Khusuwan, N. Kissoon, E. Kobeissi, T. Kostyanev, F. Krapp, R. Krumkamp, A. Kumar, H. H. Kyu, C. Lim, K. Lim, D. Limmathurotsakul, M. J. Loftus, M. Lunn, J. Ma, A. Manoharan, F. Marks, J. May, M. Mayxay, N. Mturi, T. Munera-Huertas, P. Musicha, L. A. Musila, M. M. Mussi-Pinhata, R. N. Naidu, T. Nakamura, R. Nanavati, S. Nangia, P. Newton, C. Ngoun, A. Novotney, D. Nwakanma, C. W. Obiero, T. J. Ochoa, A. Olivas-Martinez, P. Olliaro, E. Ooko, E. Ortiz-Brizuela, P. Ounchanum, G. D. Pak, J. L. Paredes, A. Y. Peleg, C. Perrone, T. Phe, K. Phommasone, N. Plakkal, A. Ponce-de-Leon, M. Raad, T. Ramdin, S. Rattanavong, A. Riddell, T. Roberts, J. V. Robotham, A. Roca, V. D. Rosenthal, K. E. Rudd, N. Russell, H. S. Sader, W. Saengchan, J. Schnall, J. A. G. Scott, S. Seekaew, M. Sharland, M. Shivamallappa, J. Sifuentes-Osornio, A. J. Simpson, N. Steenkeste, A. J. Stewardson, T. Stoeva, N. Tasak, A. Thaiprakong, G. Thwaites, C. Tigoi, C. Turner, P. Turner, H. R. van Doorn, S. Velaphi, A. Vongpradith, M. Vongsouvath, H. Vu, T. Walsh, J. L. Walson, S. Waner, T. Wangrangsimakul, P. Wannapinij, T. Wozniak, T. E. M. W. Young Sharma, K. C. Yu, P. Zheng, B. Sartorius, A. D. Lopez, A. Stergachis, C. Moore, C. Dolecek, M. Naghavi; Antimicrobial Resistance Collaborators, Global burden of bacterial antimicrobial resistance in 2019: A systematic analysis. *Lancet* **399**, 629–655 (2022). doi:10.1016/S0140-6736(21)02724-0 Medline

42. M. R. McLaren, B. J. Callahan, Pathogen resistance may be the principal evolutionary advantage provided by the microbiome. *Philos. Trans. R. Soc. London Ser. B* **375**, 20190592 (2020). doi:10.1098/rstb.2019.0592 Medline

43. M. J. Blaser, The theory of disappearing microbiota and the epidemics of chronic diseases. *Nat. Rev. Immunol.* **17**, 461–463 (2017). doi:10.1038/nri.2017.77 Medline

44. M. Fassarella, E. E. Blaak, J. Penders, A. Nauta, H. Smidt, E. G. Zoetendal, Gut microbiome stability and resilience: Elucidating the response to perturbations in order to modulate gut health. *Gut* **70**, 595–605 (2021). doi:10.1136/gutjnl-2020-321747 Medline

45. H. C. Wastyk, G. K. Fragiadakis, D. Perelman, D. Dahan, B. D. Merrill, F. B. Yu, M. Topf, C. G. Gonzalez, W. Van Treuren, S. Han, J. L. Robinson, J. E. Elias, E. D. Sonnenburg, C. D. Gardner, J. L. Sonnenburg, Gut-microbiota-targeted diets modulate human immune status. *Cell* **184**, 4137–4153.e14 (2021). doi:10.1016/j.cell.2021.06.019 Medline

46. E. Le Chatelier, T. Nielsen, J. Qin, E. Prifti, F. Hildebrand, G. Falony, M. Almeida, M. Arumugam, J.-M. Batto, S. Kennedy, P. Leonard, J. Li, K. Burgdorf, N. Grarup, T. Jørgensen, I. Brandslund, H. B. Nielsen, A. S. Juncker, M. Bertalan, F. Levenez, N. Pons, S. Rasmussen, S. Sunagawa, J. Tap, S. Tims, E. G. Zoetendal, S. Brunak, K. Clément, J. Doré, M. Kleerebezem, K. Kristiansen, P. Renault, T. Sicheritz-Ponten, W. M. de Vos, J.-D. Zucker, J. Raes, T. Hansen, P. Bork, J. Wang, S. D. Ehrlich, O. Pedersen, M. van de Guchte, G. Vandemeulebrouck, A. Jamet, R. Dervyn, N. Sanchez, E. Maguin, F. Haimet, Y. Winogradski, A. Cultrone, M. Leclerc, C. Juste, H. Blottière, E. Pelletier, D. LePaslier, F. Artiguenave, T. Bruls, J. Weissenbach, K. Turner, J. Parkhill, M. Antolin, C. Manichanh, F. Casellas, N. Boruel, E. Varela, A. Torrejon, F. Guarner, G. Denariaz, M. Derrien, J. E. T. van Hylckama Vlieg, P. Veiga, R. Oozeer, J. Knol, M. Rescigno, C. Brechot, C. M'Rini, A. Mérieux, T. Yamada, P. Bork, J. Wang, S. D. Ehrlich, O. Pedersen, E. Guedon, C. Delorme, S. Layec, G. Khaci, M. van de Guchte, G. Vandemeulebrouck, A. Jamet, R. Dervyn, N. Sanchez, E. Maguin, F. Haimet, Y. Winogradski, A. Cultrone, M. Leclerc, C. Juste, H. Blottière, E. Pelletier, D. LePaslier, F. Artiguenave, T. Bruls, J. Weissenbach, K. Turner, J. Parkhill, M. Antolin, C. Manichanh, F. Casellas, N. Boruel, E. Varela, A. Torrejon, F. Guarner, G. Denariaz, M. Derrien, J. E. T. van H. Vlieg, P. Veiga, R. Oozeer, J. Knol, M. Rescigno, C. Brechot, C. M'Rini, A. Mérieux, T. Yamada, MetaHIT consortium, Richness of human gut microbiome correlates with metabolic markers. *Nature* **500**, 541–546 (2013).

47. S. E. Winter, P. Thiennimitr, M. G. Winter, B. P. Butler, D. L. Huseby, R. W. Crawford, J. M. Russell, C. L. Bevins, L. G. Adams, R. M. Tsolis, J. R. Roth, A. J. Bäumler, Gut inflammation provides a respiratory electron acceptor for Salmonella. *Nature* **467**, 426–429 (2010). doi:10.1038/nature09415 Medline

48. K. M. Pruss, J. L. Sonnenburg, C. difficile exploits a host metabolite produced during toxin-mediated disease. *Nature* **593**, 261–265 (2021). doi:10.1038/s41586-021-03502-6 Medline

49. L. Maier, R. Vyas, C. D. Cordova, H. Lindsay, T. S. B. Schmidt, S. Brugiroux, B. Periaswamy, R. Bauer, A. Sturm, F. Schreiber, C. von Mering, M. D. Robinson, B. Stecher, W.-D. Hardt, Microbiota-derived hydrogen fuels Salmonella typhimurium invasion of the gut ecosystem. *Cell Host Microbe* **14**, 641–651 (2013). doi:10.1016/j.chom.2013.11.002 Medline

50. M. L. Jenior, J. L. Leslie, V. B. Young, P. D. Schloss, *Clostridium difficile* Colonizes Alternative Nutrient Niches during Infection across Distinct Murine Gut Microbiomes. *mSystems* **2**, e00063–17 (2017). doi:10.1128/mSystems.00063-17 Medline

51. K. M. Ng, J. A. Ferreyra, S. K. Higginbottom, J. B. Lynch, P. C. Kashyap, S. Gopinath, N. Naidu, B. Choudhury, B. C. Weimer, D. M. Monack, J. L. Sonnenburg, Microbiota-liberated host sugars facilitate post-antibiotic expansion of enteric pathogens. *Nature* **502**, 96–99 (2013). doi:10.1038/nature12503 Medline

52. A. W. Hudson, A. J. Barnes, A. S. Bray, D. A. Ornelles, M. A. Zafar, Klebsiella pneumoniae l-Fucose Metabolism Promotes Gastrointestinal Colonization and Modulates Its Virulence Determinants. *Infect. Immun.* **90**, e0020622 (2022). doi:10.1128/iai.00206-22 Medline

53. A. G. Jimenez, M. Ellermann, W. Abbott, V. Sperandio, Diet-derived galacturonic acid regulates virulence and intestinal colonization in enterohaemorrhagic Escherichia coli and Citrobacter rodentium. *Nat. Microbiol.* **5**, 368–378 (2020). doi:10.1038/s41564-019-0641-0 Medline

54. F. C. Pereira, K. Wasmund, I. Cobankovic, N. Jehmlich, C. W. Herbold, K. S. Lee, B. Sziranyi, C. Vesely, T. Decker, R. Stocker, B. Warth, M. von Bergen, M. Wagner, D. Berry, Rational design of a microbial consortium of mucosal sugar utilizers reduces Clostridiodes difficile colonization. *Nat. Commun.* **11**, 5104 (2020). doi:10.1038/s41467-020-18928-1 Medline

55. E. Gül, A. Abi Younes, J. Huuskonen, C. Diawara, B. D. Nguyen, L. Maurer, E. Bakkeren, W.-D. Hardt, Differences in carbon metabolic capacity fuel co-existence and plasmid transfer between Salmonella strains in the mouse gut. *Cell Host Microbe* **31**, 1140–1153.e3 (2023). doi:10.1016/j.chom.2023.05.029 Medline

56. N. Raffelsberger, M. A. K. Hetland, K. Svendsen, L. Småbrekke, I. H. Löhr, L. L. E. Andreassen, S. Brisse, K. E. Holt, A. Sundsfjord, Ø. Samuelsen, K. Gravningen, Gastrointestinal carriage of Klebsiella pneumoniae in a general adult population: A cross-sectional study of risk factors and bacterial genomic diversity. *Gut Microbes* **13**, 1939599 (2021). doi:10.1080/19490976.2021.1939599 Medline

57. 57. T. Mäklin, T., H. A. Thorpe, A. K. Pöntinen, R. A. Gladstone, Y. Shao, M. Pesonen, A. McNally, P. J. Johnsen, Ø. Samuelsen, T. D. Lawley, A. Honkela, J. Corander, Strong pathogen competition in neonatal gut colonisation. *Nat Commun* **13**, 7417 (2022). doi:10.1038/s41467-022-35178-5

58. S. K. Hoiseth, B. A. Stocker, Aromatic-dependent Salmonella typhimurium are non-virulent and effective as live vaccines. *Nature* **291**, 238–239 (1981). doi:10.1038/291238a0 Medline

59. L. Maier, M. Pruteanu, M. Kuhn, G. Zeller, A. Telzerow, E. E. Anderson, A. R. Brochado, K. C. Fernandez, H. Dose, H. Mori, K. R. Patil, P. Bork, A. Typas, Extensive impact of non-antibiotic drugs on human gut bacteria. *Nature* **555**, 623–628 (2018). doi:10.1038/nature25979 Medline

60. M. Tramontano, S. Andrejev, M. Pruteanu, M. Klünemann, M. Kuhn, M. Galardini, P. Jouhten, A. Zelezniak, G. Zeller, P. Bork, A. Typas, K. R. Patil, Nutritional preferences

of human gut bacteria reveal their metabolic idiosyncrasies. *Nat. Microbiol.* **3**, 514–522 (2018). doi:10.1038/s41564-018-0123-9 Medline

61. F. R. Cianfanelli, O. Cunrath, D. Bumann, Efficient dual-negative selection for bacterial genome editing. *BMC Microbiol.* **20**, 129 (2020). doi:10.1186/s12866-020-01819-2 Medline

62. R. Soldan, N. Sanguankiattichai, M. Bach-Pages, I. Bervoets, W. E. Huang, G. M. Preston, From macro to micro: A combined bioluminescence-fluorescence approach to monitor bacterial localization. *Environ. Microbiol.* **23**, 2070–2085 (2021). doi:10.1111/1462-2920.15296 Medline

63. A. M. Eren, E. Kiefl, A. Shaiber, I. Veseli, S. E. Miller, M. S. Schechter, I. Fink, J. N. Pan, M. Yousef, E. C. Fogarty, F. Trigodet, A. R. Watson, Ö. C. Esen, R. M. Moore, Q. Clayssen, M. D. Lee, V. Kivenson, E. D. Graham, B. D. Merrill, A. Karkman, D. Blankenberg, J. M. Eppley, A. Sjödin, J. J. Scott, X. Vázquez-Campos, L. J. McKay, E. A. McDaniel, S. L. R. Stevens, R. E. Anderson, J. Fuessel, A. Fernandez-Guerra, L. Maignien, T. O. Delmont, A. D. Willis, Community-led, integrated, reproducible multi-omics with anvi'o. *Nat. Microbiol.* **6**, 3–6 (2021). doi:10.1038/s41564-020-00834-3 Medline

64. I. Letunic, P. Bork, Interactive Tree Of Life (iTOL) v5: An online tool for phylogenetic tree display and annotation. *Nucleic Acids Res.* **49**, W293–W296 (2021). doi:10.1093/nar/gkab301 Medline

65. O. Cunrath, D. Bumann, Host resistance factor SLC11A1 restricts *Salmonella* growth through magnesium deprivation. *Science* **366**, 995–999 (2019). doi:10.1126/science.aax7898 Medline

66. S. Hapfelmeier, B. Stecher, M. Barthel, M. Kremer, A. J. Müller, M. Heikenwalder, T. Stallmach, M. Hensel, K. Pfeffer, S. Akira, W.-D. Hardt, The Salmonella pathogenicity island (SPI)-2 and SPI-1 type III secretion systems allow Salmonella serovar typhimurium to trigger colitis via MyD88-dependent and MyD88-independent mechanisms. *J. Immunol.* **174**, 1675–1685 (2005). doi:10.4049/jimmunol.174.3.1675 Medline

67. N. L. Sternberg, R. Maurer, Bacteriophage-mediated generalized transduction in Escherichia coli and Salmonella typhimurium. *Methods Enzymol.* **204**, 18–43 (1991). doi:10.1016/0076-6879(91)04004-8 Medline

68. A. R. Wattam, J. J. Davis, R. Assaf, S. Boisvert, T. Brettin, C. Bun, N. Conrad, E. M. Dietrich, T. Disz, J. L. Gabbard, S. Gerdes, C. S. Henry, R. W. Kenyon, D. Machi, C. Mao, E. K. Nordberg, G. J. Olsen, D. E. Murphy-Olson, R. Olson, R. Overbeek, B. Parrello, G. D. Pusch, M. Shukla, V. Vonstein, A. Warren, F. Xia, H. Yoo, R. L. Stevens, Improvements to PATRIC, the all-bacterial Bioinformatics Database and Analysis Resource Center. *Nucleic Acids Res.* **45**, D535–D542 (2017). doi:10.1093/nar/gkw1017 Medline

69. J. J. Davis, S. Gerdes, G. J. Olsen, R. Olson, G. D. Pusch, M. Shukla, V. Vonstein, A. R. Wattam, H. Yoo, PATtyFams: Protein Families for the Microbial Genomes in the PATRIC Database. *Front. Microbiol.* **7**, 118 (2016). doi:10.3389/fmicb.2016.00118 Medline

70. R Development Core Team: R: A Language and Environment for Statistical Computing (R Foundation for Statistical Computing, 2021). https://www.R-project.org/.

71. F. Spragge, E. Bakkeren, M. T. Jahn, E. B. N. Araujo, C. F. Pearson, X. Wang, L. Pankhurst, O. Cunrath, K. R. Foster, Microbiome diversity protects against pathogens by nutrient blocking, Dryad (2023); https://doi.org/10.5061/dryad.pnvx0k6v8.

72. B. Periaswamy, L. Maier, V. Vishwakarma, E. Slack, M. Kremer, H. L. Andrews-Polymenis, M. McClelland, A. J. Grant, M. Suar, W.-D. Hardt, Live attenuated S. Typhimurium vaccine with improved safety in immuno-compromised mice. *PLOS ONE* **7**, e45433 (2012). doi:10.1371/journal.pone.0045433 Medline

73. J. S. Johnson, D. J. Spakowicz, B.-Y. Hong, L. M. Petersen, P. Demkowicz, L. Chen, S. R. Leopold, B. M. Hanson, H. O. Agresta, M. Gerstein, E. Sodergren, G. M. Weinstock, Evaluation of 16S rRNA gene sequencing for species and strain-level microbiome analysis. *Nat. Commun.* **10**, 5029 (2019). doi:10.1038/s41467-019-13036-1 Medline

74. T. Matsuki, K. Watanabe, J. Fujimoto, Y. Miyamoto, T. Takada, K. Matsumoto, H. Oyaizu, R. Tanaka, Development of 16S rRNA-gene-targeted group-specific primers for the detection and identification of predominant bacteria in human feces. *Appl. Environ. Microbiol.* **68**, 5445–5451 (2002). doi:10.1128/AEM.68.11.5445-5451.2002 Medline
